# Bond-length distributions for ions bonded to oxygen: results for the non-metals and discussion of lone-pair stereoactivity and the polymerization of PO_4_

**DOI:** 10.1107/S2052520617017541

**Published:** 2018-01-13

**Authors:** Olivier Charles Gagné, Frank Christopher Hawthorne

**Affiliations:** a Geological Sciences, University of Manitoba, 125 Dysart Road, Winnipeg, Manitoba R3T 2N2, Canada

**Keywords:** bond lengths, non-metals, lone-pair stereoactivity, hydrogen, phosphate, polymerization, oxides, oxysalts

## Abstract

Bond-length distributions are examined for three configurations of the H^+^ ion, 16 configurations of the group 14–16 non-metal ions and seven configurations of the group 17 ions bonded to oxygen, for 223 coordination polyhedra and 452 bond distances for the H^+^ ion, 5957 coordination polyhedra and 22 784 bond distances for the group 14–16 non-metal ions, and 248 coordination polyhedra and 1394 bond distances for the group 17 non-metal ions. H⋯O and O—H + H⋯O distances correlate with O⋯O distance (*R* ^2^ = 0.94 and 0.96): H⋯O = 1.273 × O⋯O – 1.717 Å; O—H + H⋯O = 1.068 × O⋯O – 0.170 Å. These equations may be used to locate the hydrogen atom more accurately in a structure refined by X-ray diffraction. For non-metal elements that occur with lone-pair electrons, the most observed state between the *n* *versus* *n*+2 oxidation state is that of highest oxidation state for period 3 cations, and lowest oxidation state for period 4 and 5 cations when bonded to O^2−^. Observed O—*X*—O bond angles indicate that the period 3 non-metal ions P^3+^, S^4+^, Cl^3+^ and Cl^5+^ are lone-pair seteroactive when bonded to O^2−^, even though they do not form secondary bonds. There is no strong correlation between the degree of lone-pair stereoactivity and coordination number when including secondary bonds. There is no correlation between lone-pair stereoactivity and bond-valence sum at the central cation. In synthetic compounds, PO_4_ polymerizes *via* one or two bridging oxygen atoms, but not by three. Partitioning our PO_4_ dataset shows that multi-modality in the distribution of bond lengths is caused by the different bond-valence constraints that arise for O_br_ = 0, 1 and 2. For strongly bonded cations, *i.e.* oxyanions, the most probable cause of mean bond length variation is the effect of structure type, *i.e.* stress induced by the inability of a structure to follow its *a priori* bond lengths. For ions with stereoactive lone-pair electrons, the most probable cause of variation is bond-length distortion.

## Abstract 2

Bond-length distributions are examined for three configurations of the H^+^ ion, 16 configurations of the group 14–16 non-metal ions and seven configurations of the group 17 ions bonded to oxygen. Lone-pair stereoactivity for ions bonded to O^2−^ is discussed, as well as the polymerization of the PO_4_ group.

## 1. Introduction   

A large number of inorganic crystal structures have been refined to relatively high degrees of accuracy and precision in the past few decades. A few studies have looked at the crystal-chemical behaviour of specific ions *via* the study of their bond-length distributions, and these were listed in the first paper of this series (Gagné & Hawthorne, 2016 ▸). However, a comprehensive examination of the variation in interatomic distances of ions has yet to be done for inorganic crystal structures, despite the pivotal influence that these kinds of studies have played in organic and organometallic chemistry (*e.g.* Allen *et al.*, 1987 ▸; Mayer, 1988 ▸; Orpen *et al.*, 1989 ▸). It is the goal of this series to lay the foundation for a comprehensive examination of variation of bond lengths and bond strengths for all ion configurations bonded to oxygen, and to provide easy access to the wealth of structural data that we have gathered. The examination of these distributions also serves to verify our understanding of bonding in inorganic crystal structures, and the various structural and electronic effects that manifest themselves *via* variations in bond lengths.

We have examined the distribution of bond lengths for 135 ions bonded to oxygen in 462 configurations using 180 331 bond lengths extracted from 9367 refined crystal structures (Gagné & Hawthorne, 2016 ▸, 2018 ▸; Gagné, 2018 ▸); these data involve most ions of the periodic table and all coordination numbers in which they occur. In the present work, we report the bond-length distributions for 16 non-metal ions bonded to O^2−^: for H^+^ in three configurations (*n* = 452 bond lengths and 223 coordination polyhedra from 68 crystal-structure refinements using neutron diffraction data), for eight group 14–16 non-metal ions in 16 configurations (22 784 bond lengths and 5826 coordination polyhedra from 2909 crystal-structure refinements), and for seven group 17 non-metal ions in 14 configurations (*n* = 1394 bond lengths and 248 coordination polyhedra from 163 crystal-structure refinements). The availability and analysis of large amounts of data can (1) overcome the problem of the possible derivation of non-representative behaviour due to small datasets, and (2) allow subtle effects to become more apparent *via* comparison of data for many different ion configurations. In the first paper of this series (Gagné & Hawthorne, 2016 ▸), we reported bond-length distributions for the alkali metal ions (Li^+^, Na^+^, K^+^, Rb^+^ and Cs^+^) and alkaline earth metal ions (Be^2+^, Mg^2+^, Ca^2+^, Sr^2+^ and Ba^2+^) in all observed coordination numbers where bonded to O^2−^, and gave a detailed introduction and rationale for this work and a description of the data-collection and data-filtering methods.

## 2. Lone-pair stereoactivity   

Of the 135 ions for which we have collected data, 11 ions have lone-pair electrons that are stereoactive. As this is the first paper in our series on bond-length distributions for cations bonded to oxygen that describes such ions, here we give a general discussion on lone-pair stereoactivity and discuss different models that attempt to rationalize lone-pair stereoactivity.

Lone-pair stereoactivity is associated with pronounced asymmetry in the coordination polyhedra of *p*-block cations with *ns* ^2^ *np* ^0^ electron configurations. Early investigations of lone-pair stereoactivity include that by Sidgwick & Powell (1940 ▸), who proposed that lone-pair electrons are equivalent to bonded electron pairs in minimizing electrostatic repulsion *via* geometrical arguments. This was later modified by Gillespie & Nyholm (1957 ▸) who recognized that repulsion involving lone-pair electrons is greater than that arising from bonded electrons, leading to their development of the valence-shell electron-pair repulsion (VSEPR) model.

Orgel (1959 ▸) described the origins of the stereochemical behaviour of *ns* ^2^ *np* ^0^ cations based on the mixing of their non-bonding *s* and *p* orbitals. He argued that the formation of *sp*-hybridized orbitals may only occur at non-centrosymmetric sites due to the parity constraints of these orbitals, hence, the observation of distorted structures for many cations with lone-pair electrons. Orgel described these ions as coordinated by three or four short bonds in one hemisphere, typically with intermediate (2.4–2.6 Å) to long (2.6–3.1 Å) bonds in the other hemisphere. Following this, Durrant & Durrant (1962 ▸) provided separate definitions for ‘inert’ and ‘stereoactive’ lone-pair electrons: *inert lone pairs* are those that remain in the original atomic orbitals of the cation and do not engage in orbital hybridization, and *stereoactive lone pairs* are those for which the original atomic orbital is involved in hybridization where one of the hybrid orbitals becomes occupied by the lone-pair electrons. Stereoactive and inert lone pairs are also called by other names today, *e.g.* stereochemically active and stereochemically inactive (Pyykkö, 1988 ▸), and hemi-directed and holo-directed (Shimoni-Livny *et al.*, 1998 ▸).

Galy *et al.* (1975 ▸) studied 12 ions with lone-pair electrons from groups 13–18 (Ge^2+^, As^3+^, Se^4+^, Br^5+^, Sn^2+^, Sb^3+^, Te^4+^, I^5+^, Xe^6+^, Tl^+^, Pb^2+^, Bi^3+^) in an effort to rationalize their stereochemical behaviour. They described a decrease in lone-pair stereoactivity down and left in the periodic table of elements, and found that only Br^5+^ and Xe^6+^ have fully stereoactive lone-pair electrons, whereas other ions are observed in intermediate states. As a result of this, they suggest the term *lone-pair electrons* as a more suitable and generally applicable alternative to the inert and stereoactive lone-pair terminology of Durrant & Durrant (1962 ▸). Galy *et al.* (1975 ▸) also suggested that the hybrid orbital containing the stereoactive lone-pair electrons is similar in size to their orbital where they are bonded to an anion, and provided a formula for calculating the degree of stereoactivity of the cation based on the distance between the cation and its lone-pair electrons, as discussed by Andersson & Åström (1972 ▸). They also discussed the bond geometry of compounds containing lone-pair stereoactive electrons, coming to a conclusion similar to that of Gillespie & Nyholm (1957 ▸) and Gillespie (1972 ▸) who used the valence-shell electron-pair repulsion (VSEPR) theory to rationalize the geometry of compounds containing lone-pair stereoactive ions based on orbital hybridization.

Bersuker (1984 ▸) proposed that lone-pair stereoactivity is determined by the energy separation between the highest occupied molecular orbital (HOMO) of the cation and the lowest unoccupied molecular orbital (LUMO) of the anion. A number of electronic structure studies done in the following years (*e.g.* Lefebvre *et al.* 1987 ▸, 1998 ▸; Watson & Parker, 1999 ▸; Watson *et al.*, 1999 ▸; Seshadri & Hill, 2001 ▸; Waghmare *et al.*, 2003 ▸) confirmed the proposal of Bersuker. More recently, Stoltzfus *et al.* (2007 ▸) studied SnWO_4_, PbWO_4_ and BiVO_4_ using density functional theory and UV–visible diffuse reflectance spectroscopy. They showed a strong interaction between the 5*s* orbitals of the Sn^2+^ ion and the 2*p* orbitals of oxygen as having a significant destabilizing effect in symmetric structures, which may be reduced by lowering the symmetry of the Sn^2+^ site and enabling the antibonding Sn 5*s*–O 2*p* states to mix with the unfilled Sn 5*p* orbitals (a pseudo Jahn–Teller effect, where the symmetry of the distorted structure should be one that enables the Sn 5*s* and Sn 5*p* states to mix). They state that for Pb^2+^ and Bi^3+^, relativistic contraction of the 6*s* orbital reduces this interaction, thus favouring the formation of structures with higher symmetry and more diffuse lone-pair electrons.

In a review of the stereochemistry of post-transition-metal oxides, Walsh *et al.* (2011 ▸) similarly describe lone-pair stereoactivity as a pseudo Jahn–Teller effect and give a revised model of lone-pair stereoactivity with explicit dependence on the anion, which they call the revised lone-pair model. They state that strong interactions between the cation *s* and anion *p* orbitals result in a high-energy antibonding state, which, *via* distortion of the crystal structure, may interact with the empty cation *p* orbitals to form an electronic state where the lone pair resides. It is the occurrence of this favourable interaction through distortion that, for *certain* structures, results in stabilization of the occupied electronic states, leading to the stereoactivity of the lone pair. Compounds in which the energy of the anion *p* states is too large relative to the cation *s* state will have no such favourable interaction, *e.g.* the chalcogenides, and have an inert (and diffuse) lone pair. Walsh *et al.* (2011 ▸) add that in the formation of a stereoactive lone pair, the contribution of the cation *s* state to the antibonding state is crucial in creating a favourable interaction with the cation *p* state; the stronger the *s* state contribution, the stronger the stabilization of the antibonding state. The amount of *s* character carried to the antibonding state follows symmetry arguments and is, therefore, structure dependent.

### 2.1. Lone-pair stereoactivity in the bond-valence model   

The bond-valence model (Brown, 2016 ▸) is a model of chemical bonding used in inorganic chemistry as a simple and often very useful alternative to quantum theory. Here, we discuss the interpretation of lone-pair stereoactivity from the perspective of this model.

Following a review of 28 Tl^+^ compounds, Brown & Faggiani (1980 ▸) showed an inverse relation between the coordination number of Tl^+^ and the base strength of the anion. They proposed that Tl^+^ will form short bonds, have a low coordination number and a stereoactive lone pair where the counterion is a strong base (Lewis basicity > 0.22 v.u.), and will otherwise form longer bonds and have a coordination number greater than [6]. In this work, they treat the ‘base’ as the oxyanion groups, *e.g.* BO_3_^3−^, rather than individual O^2−^ ions, and define the bond strength of the base as the total negative charge of the group divided by the number of bonds it forms; this gives Lewis base strengths of 0.04–0.50 v.u. for the 28 compounds analyzed. This practice of comparing bond strength between cations and anions paved the way to the ‘valence-matching principle’ (Brown, 1981 ▸), a key feature of the bond-valence model that allows *a priori* analysis of structure stability *via* Lewis acid–base arguments. While Brown & Faggiani observed that structures with a Lewis base strength greater than 0.22 v.u. are always stereoactive, a mixture of lone-pair stereoactivity and inactivity is observed below that threshold.

Brown (1988 ▸) proposed a vector-based description of bond-length distortion to be a measure of the stereoactivity of the lone pair. He revised the Lewis base strength cut-off from 0.22 v.u. to 0.27 v.u. in discussing Tl^+^ structures (revised again in 2011 to 0.23 v.u.), and added that for bases stronger than 0.27 v.u., the bonding is fully directed and the geometry is explained by the VSEPR model (Gillespie, 1972 ▸). However, a mixture of lone-pair stereoactivity and inactivity is again observed below that threshold, and remains unexplained.

Brown (2011 ▸) points out that the VSEPR model fails to explain the many cases in which ions with lone pairs are found in high-symmetry environments, *i.e.* where the electrons are uniformly distributed around the valence shell. In those structures, Brown describes the ions concerned as behaving like main-group ions obeying the valence-matching principle, *i.e.* 0.5 < *S*_A_/*S*_B_ < 2, where *S*_A_ is the Lewis acid strength of the cation [see Gagné & Hawthorne (2017*a*) ▸ for a comprehensive list] and *S*_B_ is the Lewis base strength of the anion (or group). He explains that in those cases, ions with lone-pair electrons have an inherent flexibility to form stronger bonds by converting lone-pair electron density to bonding electron density in the region where the valence shells overlap. Bonding electron density elsewhere in the valence shell is then converted to lone pairs, which Brown states is possible due to the electrons losing their identity once inside the atom. It is also proposed that the anisotropy observed for ions with lone-pair electrons is not caused by the lone pair but rather by the concentration of bonding electron density in the region of the stronger bonds, with the lone-pair electron density merely occupying regions of the valence shell where bonds are not formed. This treatment suggests that a partial positive charge is left in the hemisphere in which the lone pair resides. This suggestion is in accord with our observations on 1000+ coordination polyhedra analyzed for ions with lone-pair electrons (this work; Gagné & Hawthorne, 2018 ▸) that the general vicinity of the lone pair is occupied by anions, generally with no interaction between the lone pair and other cations.^1^ Brown extended this idea to anions with lone-pair electrons, stating that their lone pair is similarly stereoactive only when the counterion has a large bonding strength, *e.g.* for a coordination number of [4] for O^2−^ as it approaches 2*S*_B_ = 1 v.u.

Brown (2016 ▸) states that the degree of stereoactivity observed for the lone pair depends on several factors, primarily the bonding strength of the primary ligand (*S*_A_), but also steric effects or the strength of the electric field (flux density) of the bond.

Although the level of rigorousness of the bond-valence model is nowhere near that of quantum theory when it comes to the treatment of lone-pair stereoactivity, it is useful that lone-pair stereoactivity may be predicted based purely on Lewis acid–base arguments.

## 3. Coordination number   

A contentious issue in the description of lone-pair stereoactive ions is that of coordination number. Whereas coordination number may be defined in simple terms, *e.g.* the number of counterions bonded to an ion, the decision of considering atom pairs as ‘bonded’ or not is less obvious in many situations. By-and-large, the determination of coordination number in ambiguous cases is a matter of judgement. This problem is accentuated with lone-pair stereoactive ions. For these ions, bonds are typically referred to as ‘primary’, *i.e.* short and strong, and ‘secondary’ (Alcock, 1972 ▸) or ‘tertiary’ (Preiser *et al.*, 1999 ▸), *i.e.* long and weak. For example, Brown & Faggiani (1980 ▸) included all interatomic distances up to 3.5 Å in their description of 28 Tl^+^ structures, and gave 3.1 Å as the cut-off between primary and secondary. This problem is further complicated by ‘intermediate states’ of lone-pair stereoactivity, as described by Galy *et al.* (1975 ▸). Here, we adopt the terminology of Alcock (1972 ▸) without imposing a strict cut-off between primary and secondary bonds.

Preiser *et al.* (1999 ▸) calculated the electric flux that links neighbouring ions of opposite charge in structures, which they identify as the bond valence, and describe stereoactive lone-pair electrons as creating electronic anisotropy in the structure whereby the equal-valence rule is no longer obeyed, but where the valence-sum rule still holds. They provide evidence in support of the work of Alig & Trömel (1992 ▸) that some of the longer cation–anion distances may contribute to weak but significant chemical bonding, stating that electrostatic fluxes are observed between ions separated by as much as 3–4 Å in some structures. This trend toward considering longer cation–anion distances as having significant bonding interactions continued with the more recent derivation of bond-valence parameters for the following lone-pair stereoactive ions bonded to O^2−^: Tl^+^ (Locock & Burns, 2004 ▸), Sb^3+^ (Palenik *et al.*, 2005 ▸; Sidey *et al.*, 2008 ▸; Sidey, 2009 ▸; Mills *et al.*, 2009 ▸; Krivovichev, 2012 ▸), Sn^2+^and I^5+^ (Sidey, 2006 ▸, 2009 ▸), Pb^2+^ (Krivovichev, 2012 ▸) and Te^4+^ (Sidey, 2006 ▸, 2009 ▸; Mills & Christy, 2013 ▸). In particular, Mills & Christy (2013 ▸) state that there is no essential difference in character between short primary Te—O bonds, oriented away from the Te lone pair, and longer secondary Te—O bonds on the same side of the Te atom as the lone pair.

Gagné & Hawthorne (2015 ▸, 2016 ▸) also provided arguments for including longer interatomic distances as ‘bonded’ by analyzing (1) trends in bond-valence parameters, and (2) the gap between the first and second coordination shell. For (1), they derived bond-valence parameters both including and excluding the longer interatomic distances. Whereas these parameters showed the same agreement for bond-valence sums, as long as they are used in the way they are derived, the exclusion of the longer interatomic distances in the coordination polyhedron leads to the loss of trends in the bond-valence parameter *R*_o_. For (2), they analyzed the significance of the gap between the bonds of the first coordination shell ([CN]) and the shortest distance to the second coordination shell (+1), comparing the resulting gap between coordinations [3]+1 and [4], [8]+1 and [9], and [13]+1 and [14]. They show a trend whereby the gap between [CN]+1 and [CN+1] decreases as [CN] increases, where the determination of the longest bond becomes more ambiguous, but also show that their method is reliable and consistent where bonds are gathered for the first coordination shell.

The rigorous method described by Gagné & Hawthorne (2016 ▸), which favours the inclusion of the longer interatomic distances in the first coordinations shell, was followed in this work to derive the first coordination shells of ions with lone-pair electrons.

## 4. Sample size   

A critical issue involved in the calculation of the grand mean bond length, skewness and kurtosis values of bond-length distributions is whether the sample size is sufficiently large to ensure a representative distribution. In the first paper of this series (Gagné & Hawthorne, 2016 ▸), we described the effects of sampling (*e.g.* the presence of outliers, non-random sampling) and of sample size on grand mean bond length (and its standard deviation), skewness, and kurtosis for the alkali and alkaline earth metal ions bonded to O^2−^. As the current work deals with ions with dramatically different crystal chemistry, we report a similar analysis for ^[4]^S^6+^ and ^[6]^I^5+^.

**Figure 1:**
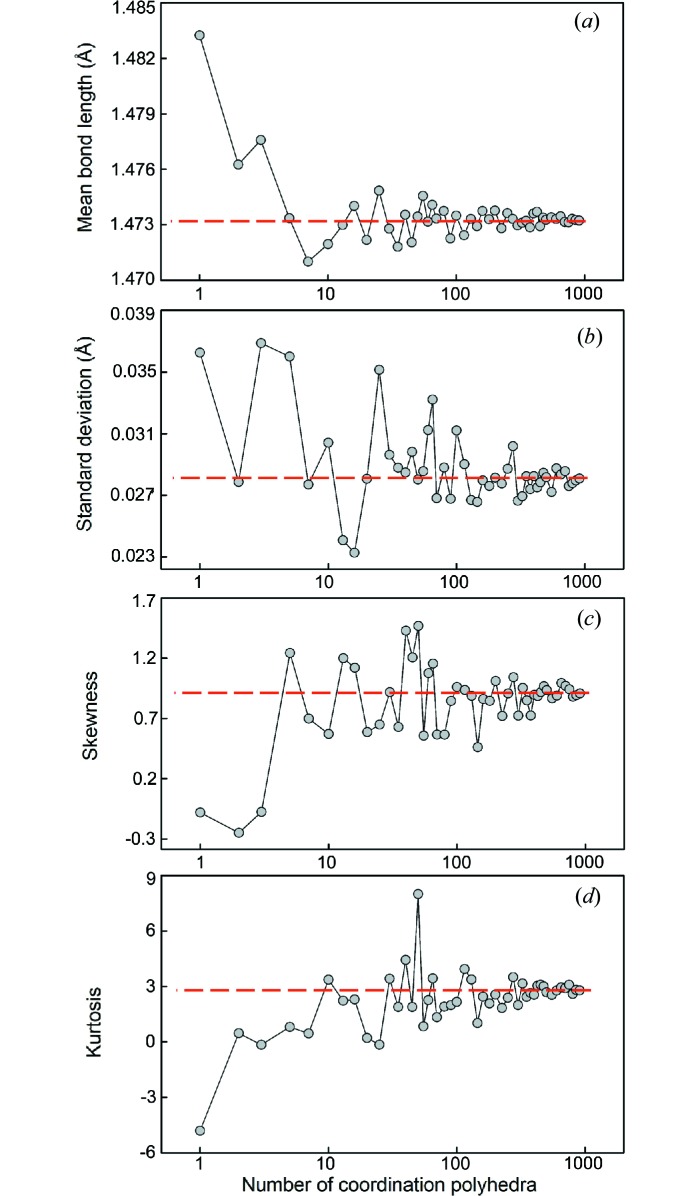
The effect of sample size on (*a*) mean bond length, (*b*) standard deviation of the mean bond length, (*c*) skewness and (*d*) kurtosis for ^[4]^S^6+^. The dashed line shows the value for the parent distribution.

We have calculated the grand mean bond length (and associated standard deviation), skewness and kurtosis for different sample sizes of coordination polyhedra randomly selected from the parent distribution for ^[4]^S^6+^ and for ^[6]^I^5+^, and report the results in Figs. 1 ▸ and 2 ▸. We report sample size as a function of the number of coordination polyhedra [sample size was reported as number of bonds by Gagné & Hawthorne (2016 ▸)].

**Figure 2:**
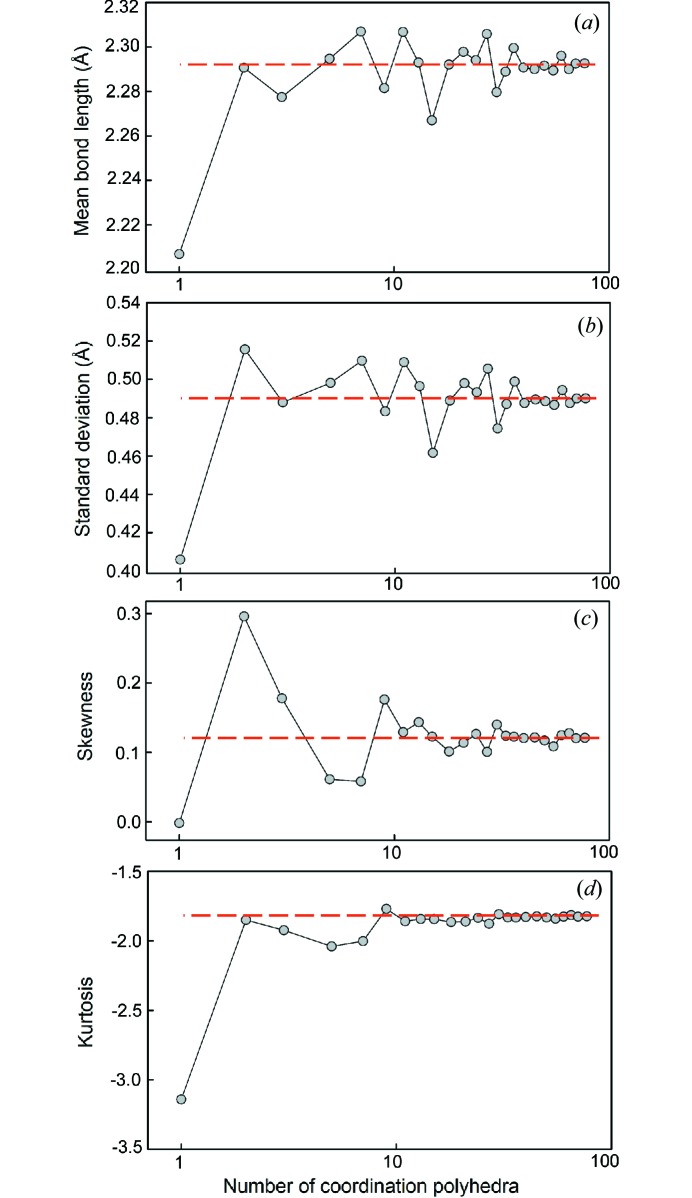
The effect of sample size on (*a*) mean bond length, (*b*) standard deviation of the mean bond length, (*c*) skewness and (*d*) kurtosis for ^[6]^I^5+^. The dashed line shows the value for the parent distribution.

Fig. 1 ▸ shows that for ^[4]^S^6+^, a reliable estimate of the grand mean bond length may be obtained from as little as five coordination polyhedra, above which variability remains below ∼±0.005 Å. However, fairly reliable values of skewness (±0.2) and kurtosis (±0.6) are only obtained for sample sizes greater than 300 coordination polyhedra; this is due to the long tail of the distribution and the effect of including relatively long bond lengths in the calculation of skewness and kurtosis. For 10–300 coordination polyhedra, skewness values vary by ∼±0.5 whereas kurtosis varies by ∼±5, showing that the issue raised by Gagné & Hawthorne (2016 ▸) for these values (discussing ^[6]^Na^+^) is also relevant to strongly bonded cations. Fig. 2 ▸ shows the opposite for ^[6]^I^5+^, whereby reliable values of skewness and kurtosis are obtained for as few as two coordination polyhedra, whereas grand mean bond lengths do not stabilize to variation of less than ±0.005 Å until a sample size of 40 coordination polyhedra is reached. In comparison, ^[6]^Na^+^ showed variation of less than ±0.005 Å for sample sizes greater than 200 coordination polyhedra and variations of less than ±0.2 and ±0.6, respectively, for samples greater than 225 coordination polyhedra.

From these two plots, we conclude that (1) strongly bonded cations require little data (approximately five coordination polyhedra) for a reasonably accurate estimation of grand mean bond length, because these ions have very little variability in their observed mean bond lengths, and (2) values of skewness and kurtosis can be calculated accurately with relatively small amounts of data (approximately five coordination polyhedra) for ions with lone-pair electrons, as the variation observed between coordination polyhedra of different structures has very little effect on these values due to the overwhelming effect of the gap between the primary and secondary bonds.

For the description of mean bond length distributions, minimum sample sizes were determined for skewness and kurtosis with the same cut-offs as above, less than which these values have little significance and are not given. For ^[4]^S^6+^, the threshold was observed at ∼700 coordination polyhedra, and for ^[6]^I^5+^, ∼50 coordination polyhedra. Gagné & Hawthorne (2016 ▸) found significant variability for values of skewness and kurtosis for sample sizes below 100 coordination polyhedra for ^[6]^Na^+^; following the cut-offs given here, reliable values of skewness and kurtosis are obtained for samples greater than 400 coordination polyhedra.

## 5. Results   

Here, we give the bond-length distributions for 16 non-metals ions bonded to O^2−^ observed in our bond-length dispersion analysis of inorganic structures, and give bond-length statistics for each ion as a function of coordination number. We noticed that the bond-length values at the tails of the distributions tend to involve a disproportionately large number of highly absorbing compounds (*i.e.* containing U, Pb, *etc*.). This behaviour (even more exaggerated) is characteristic of early structure determinations in the 1930s when no absorption corrections were done, and we suspect that the present disproportionate occurrence of very short and very long bond lengths in these compounds is due to either (1) inaccurate absorption corrections, or (2) total attenuation of the X-ray beam along the longer transmission paths through a crystal. Thus we examined heavily absorbing structures in the tails particularly carefully to check that the bond-valence sums were reasonable and that there were no anomalously large *U*_eq_ values for any of the constituent ions; structures that showed such anomalous values were discarded.

### 5.1. Hydrogen   

The positional parameters for H derived from X-ray data show significant systematic error. The electron density notionally associated with the H atom is partly delocalized into the O—H bond, leading to O—H distances that are shorter than the corresponding internuclear O—H distances and experimental H⋯O (hydrogen-bond) distances that are systematically longer than the H⋯O internuclear distances. In order to avoid this problem, we have considered H only in structures refined from neutron diffraction data.

**Figure 3:**
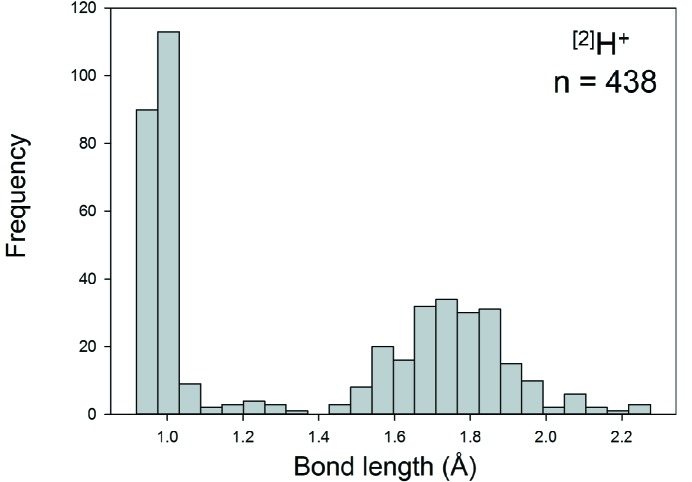
Bond-length distribution for ^[2]^H^+^ bonded to O^2−^.

Collection and filtering criteria described by Gagné & Hawthorne (2016 ▸) resulted in a sample size of 452 bonds and 223 coordination polyhedra. Where bonded to O^2−^, we observe the H^+^ ion in coordination numbers [2], [3] and [4]. Table 1 ▸ gives the bond-length distribution statistics for the three configurations, and Fig. 3 ▸ shows the bond-length distribution for coordination number [2]. All bond-length and bond-valence distributions for H^+^—O^2−^ [using the bond-valence parameters reported by Gagné & Hawthorne (2015 ▸)] are shown in Figs. S1 and S2 (supporting information), respectively. As will be discussed below for PO_4_, the bond-valence distributions are very useful in analyzing the bonding pattern of ion pairs.

**Figure 4:**
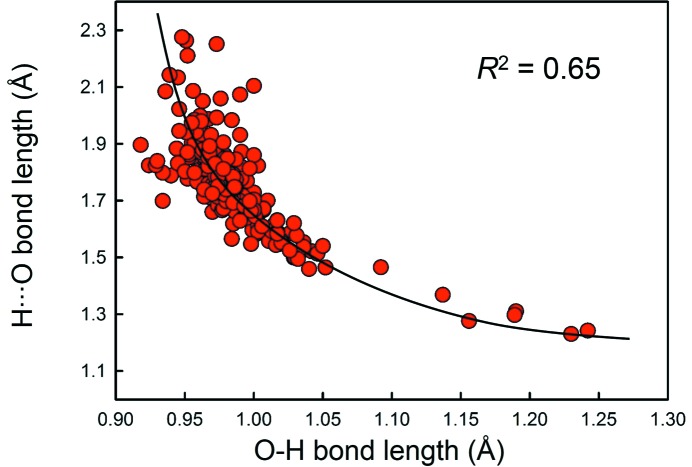
Variation of the H⋯O(acceptor) hydrogen bond distance as a function of the O(donor)—H distance for ^[2]^H^+^. The solid line shows accord with the valence-sum rule for the bond-valence parameters given by Gagné & Hawthorne (2015 ▸).

For ^[2]^H^+^ (*n* = 219), the distribution is predominantly bi-modal as expected: the left-hand distribution is for O_donor_—H bonds with a mean length of 0.983 Å, a standard deviation of 0.028 Å and a range of 0.918–1.137 Å, and the other is for H⋯O_acceptor_ bonds with a mean length of 1.764 Å, a standard deviation of 0.156 Å and a range of 1.368–2.275 Å. We also observe a small maximum at 1.236 Å in Fig. 3 ▸; this value corresponds approximately to the length of a symmetrical hydrogen bond, and has a bond valence of 0.48 v.u. [calculated using the bond-valence parameters of Gagné & Hawthorne (2015 ▸)]. Fig. 4 ▸ shows the variation in the length of the H⋯O(acceptor) hydrogen bond as a function of the O(donor)—H distance for ^[2]^H^+^; the solid line shows accord with the valence-sum rule (Brown, 2016 ▸) using the H^+^—O^2−^ bond-valence parameters of Gagné & Hawthorne (2015 ▸). This figure shows that the H^+^—O^2−^ interaction may be modelled by a single set of bond-valence parameters across the whole range of observed distances.

**Figure 5:**
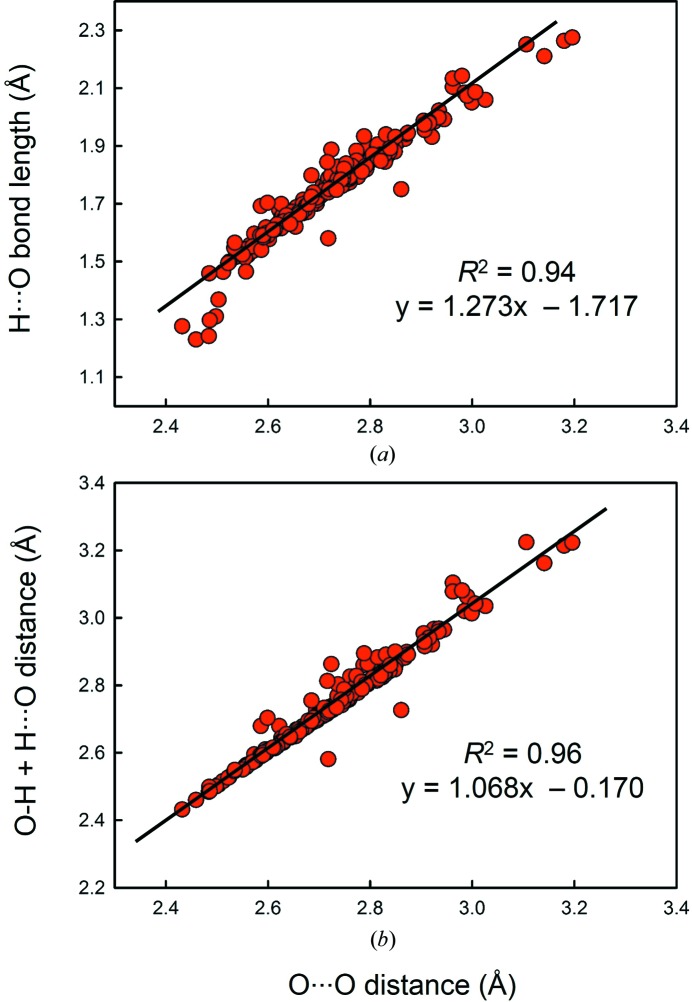
Relation between (*a*) H⋯O, (*b*) O—H + H⋯O and O⋯O distance.

**Figure 6:**
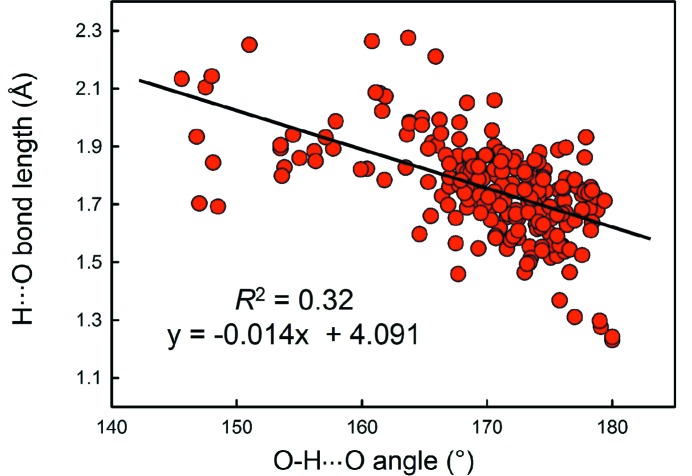
Relation between O⋯H distance and O—H⋯O angle.

Correlation of O—H, H⋯O and the sum of these two distances as a function of O⋯O distance gave *R* ^2^ = 0.38, 0.94 and 0.96, respectively. In Fig. 5 ▸, we give the relation between (*a*) H⋯O and (*b*) O—H + H⋯O *versus* O⋯O distance. The best-fit equations are (*a*) H⋯O = 1.273 × O⋯O − 1.717 Å and (*b*) O—H + H⋯O = 1.068 × O⋯O − 0.170 Å. These equations may be used to locate the hydrogen atom more accurately in a structure refined by X-ray diffraction, where the O⋯O distance may be used to position the H atom at the intersection of the O—H vector and a sphere drawn around the acceptor O atom with radius predicted by (*a*). In Fig. 6 ▸, we give a correlation between O⋯H and O—H⋯O angle (*R* ^2^ = 0.32, *p*-value 5 × 10^−20^).

Unlike organic crystals, *e.g.* carbohydrates, amino acids and proteins, for which over 25% of O—H⋯O hydrogen bonds are ‘multi-furcated’ (Steiner, 2002 ▸), multi-furcated hydrogen bonds are not common in inorganic solids. There are only a small number of examples of bifurcated and trifurcated hydrogen bonds in our neutron dataset: ^[3]^H^+^ (*n* = 2) and ^[4]^H^+^ (*n* = 2). In each case, the mean O_donor_—H bond length is slightly longer and the mean H⋯O_acceptor_ distance is significantly longer than is the case for the configuration with a single hydrogen bond (Table 1 ▸).

### 5.2. Group 14–16 non-metals   

**Figure 7:**
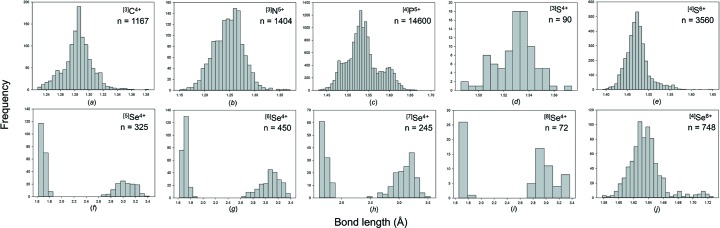
Bond-length distributions for selected configurations of the group 14–16 non-metal ions bonded to O^2−^: (*a*) ^[3]^C^4+^, (*b*) ^[3]^N^5+^, (*c*) ^[4]^P^5+^, (*d*) ^[3]^S^4+^, (*e*) ^[4]^S^6+^, (*f*) ^[5]^Se^4+^, (*g*) ^[6]^Se^4+^, (*h*) ^[7]^Se^4+^, (*i*) ^[8]^Se^4+^, (*j*) ^[4]^Se^6+^.

We obtain a combined sample size of 22 784 bonds and 5826 coordination polyhedra for 16 configurations of the group 14–16 non-metals bonded to O^2−^. Table 2 ▸ gives the bond-length statistics for all configurations, and Fig. 7 ▸ shows the bond-length distributions for sample sizes deemed significant from the results of our sample size study (above). All bond-length and bond-valence distributions for group 14–16 non-metals bonded to O^2−^ are shown in Figs. S3 and S4.

#### 5.2.1. C^4+^   

C^4+^ occurs only in one coordination: [3]. The variation in bond length is very symmetrical about its grand mean value of 1.284 Å; skewness = 0.1 (Table 2 ▸). The shortest confirmed bond length is ∼1.228 Å in sheldrickite (Grice *et al.*, 1997 ▸); smaller values have been recorded, but are usually associated with disordered and/or highly absorbing crystals. The longest distance is 1.384 Å in K(CH_3_)(CO_3_) (Adam & Cirpus, 1994 ▸). However, this is an anomalous environment, involving an O^2−^ ion bridging a CO_3_ group and a CH_3_ group: the C—O bond lengths are 1.384 Å and 1.428 Å with the corresponding bond valences 1.036 + 0.928 = 1.964 v.u. The longest C—O bonds occur in bicarbonate groups: CO_2_OH with C—OH distances up to 1.360 Å, with a C—O bond valence of 1.10 v.u. in accord with a strong O(donor)—H bond of ∼0.90 v.u.

#### 5.2.2. N^5+^   

N^5+^ occurs in two coordinations: [3] and [4], with [3] (*n* = 468) dominating over [4] (*n* = 3). The variation in bond length for [3] coordination is extremely symmetrical about its grand mean value of 1.247 Å, and the skewness is ∼0 (Table 2 ▸). Many very short reported bond lengths are not reliable: N^5+^—O^2−^ bond valences of > 2.5 v.u. are not uncommon, and bond-valence sums at the central N^5+^ exceed 7 v.u. in some cases. A reliable minimum value is ∼1.16 Å. Very long bond lengths often have very low incident bond-valence sums on the constituent O^2−^ ion and commonly are associated with very short N^5+^—O^2−^ bond lengths and high incident bond valences. The longest reliable N^5+^—O^2−^ bond length is 1.371 Å in (NH_4_)[Zr(NO_3_)_5_](HNO_3_) (Morozov *et al.*, 2005 ▸); this involves an acid nitrate group and the resulting bond valences are reasonable at 1.29 + 0.80 = 2.09 v.u. (taking an average O—H bond valence of 0.80 v.u.). There are only three examples of ^[4]^N^5+^ with a grand mean value of 1.385 Å and a range of 1.377 to 1.397 Å. Above, we conclude that strongly bonded cations require little data for a reasonably accurate estimation of grand mean bond length because these ions have very little variability in their observed mean bond lengths, and thus the value of 1.385 Å for the mean value of ^[4]^N^5+^—O^2−^ bonds should be reasonably accurate.

#### 5.2.3. P^3+^   

P^3+^ occurs in one coordination: [3], with *n* = 7 and a grand mean bond length of 1.536 Å. Unlike C^4+^ and N^5+^, the coordination is not triangular but markedly triangular pyramidal with the P^3+^ ion occupying the pyramidal position and the O^2−^—P^3+^—O^2−^ angles in the range ∼95–99^o^, far from the values of triangular coordinations that are centred on 120^o^. This arrangement suggests that the lone pair of electrons in P^3+^ occupies the fourth ‘tetrahedral’ vertex on the side of the P^3+^ ion opposing the P^3+^—O^2−^ bonds. It is also notable that P^3+^ always occurs together with P^5+^ in our data.

#### 5.2.4. P^5+^   

P^5+^ occurs in only one coordination: [4] with a grand mean bond length of 1.537 Å for 3691 coordination polyhedra, in exact agreement with the values found by Baur (1974 ▸) and Huminicki & Hawthorne (2002 ▸) for minerals. There is more than one maximum in the distribution; this will be discussed in more detail below. The shortest reliable P^5+^—O^2−^ distance is 1.430 Å and occurs in the structure of P_4_O_9_ (= P^3+^P^5+^_3_O_9_) (Lueer & Jansen, 1991 ▸); the constituent anion is only [1] coordinated and hence one can make the argument that this must be the shortest P^5+^—O^2−^distance possible. The shortest P^5+^—O^2−^ distance in our dataset is 1.423 Å (1.654 v.u.) for U_2_(PO_4_)(P_3_O_10_) (Podor *et al.*, 2003 ▸) resulting in an incident bond-valence sum of 2.28 v.u. at the constituent O^2−^ ion. For P—O distances slightly greater than 1.43 Å, *e.g*. 1.434 Å in K_4_Zn(P_3_O_9_)_2_·4H_2_O (Seethanen *et al.*, 1978 ▸), incident bond-valence sums are close to ideal (*e.g*. 1.61 + 0.11 × 3 = 1.94 v.u.). The longest P^5+^—O^2−^ distance reported is 1.718 Å in phurcalite, Ca_2_(UO_2_)_3_(PO_4_)_2_(OH)_4_(H_2_O)_4_ (Piret & Declercq, 1978 ▸), but a later refinement of the structure (Atencio *et al.*, 1991 ▸) listed the longest P^5+^—O^2−^ distance as 1.56 Å. The longest reliable P^5+^—O^2−^ distances are for O^2−^ ions bridging two phosphate groups. Jouini *et al.* (1984 ▸) report P^5+^—O^2−^ distances of 1.696 and 1.578 Å about a bridging O^2−^ ion in CuNa_3_(P_3_O_10_)(H_2_O)_12_ for an incident bond valence of 0.84 + 1.12 = 1.96 v.u., although no hydrogen positions are known and hence hydrogen bond valences are not included. Other P^5+^—O^2−^ distances somewhat less that this value are reported for P^5+^—O^2−^—P^5+^ arrangements in polymerized phosphate structures, *e.g*. 1.664 and 1.578 Å = 0.91 + 1.12 = 2.03 v.u. in Cs_2_Cu_7_(P_2_O_7_)_4_(CsCl)_6_ (Huang & Hwu, 2003 ▸).

#### 5.2.5. S^4+^   

S^4+^ occurs in one coordination: [3], with *n* = 30 and a grand mean bond length of 1.529 Å. As with P^3+^, the coordination is triangular pyramidal with the S^4+^ ion occupying the pyramidal position and the O^2−^—S^4+^—O^2−^ angles in the range ∼99–107°, with a mean value of ∼104°.

#### 5.2.6. S^6+^   

S^6+^ occurs in only one coordination: [4] with a grand mean bond length of 1.473 Å for 890 coordination polyhedra, identical to that found by Hawthorne *et al.* (2000 ▸) for sulfate minerals. The shortest reliable S^6+^—O^2−^ distance is ∼1.39 Å for which there are several structures, whereas shorter distances are associated with such issues as incommensurate structures [*e.g*. Ag(O_3_SOH) (Dell’Amico *et al.*, 1998 ▸)]. The longest distances occur in *M* ^+^_2_S_2_O_7_ structures in which an O^2−^ ion is bonded to two S^6+^ ions. This arrangement occurs in the structures of K_2_S_2_O_7_ (Swain & Guru Row, 2008 ▸) and Cs_2_S_2_O_7_ (Ståhl *et al.*, 2009 ▸). In K_2_S_2_O_7_, the two S^6+^—O^2−^ distances are 1.631 and 1.632 Å with bond valences of 1.008 + 1.005 = 2.013 v.u. in accord with the valence-sum rule. In Cs_2_S_2_O_7_, there are two sets of S^6+^—O^2−^ distances: 1.595 and 1.647 Å and 1.620 and 1.631 Å with incident bond valences of 1.103 + 0.968 = 2.071 v.u. and 1.036 + 1.008 = 2.044 v.u., both of which are in accord with the valence-sum rule. Thus the longest S^6+^—O^2−^ distance is 1.647 Å, specifically involved in an S^6+^—O^2−^—S^6+^ arrangement. Somewhat shorter but still unusually long S^6+^—O^2−^ distances occur in acid sulfate groups. For example, in K(SO_3_OH) (Swain & Guru Row, 2008 ▸), there are S^6+^—(OH)^−^ distances of 1.570 and 1.568 Å, with corresponding bond valences of 1.174 and 1.180 v.u. which, when combined with ideal O^2−^—H^+^ bond valences of 0.80 v.u., agree closely with the valence-sum rule. Thus S^6+^—(OH)^−^ distances of 1.570 Å are common.

#### 5.2.7. Se^4+^   

Se^4+^ occurs in seven coordination numbers from [3] to [10] with an average-observed coordination number of ∼[6], a fairly symmetrical distribution of coordination numbers about this mean value, and a grand mean bond length of 2.339 Å for 202 polyhedra. Se^4+^ is strongly lone-pair stereoactive and most of the coordination numbers show a bimodal distribution of bond lengths [Figs. 2(*f*)–2(*i*)]. For ^[3]^Se^4+^, there are no secondary bonds and the grand mean bond length is correspondingly short: 1.691 with a range of 1.639–1.752 Å. All other coordination numbers involve secondary bonds, and this is reflected in their much larger mean Se^4+^—O^2−^ bond lengths (Table 2 ▸) of 2.027–2.882 Å. Se^4+^ shows only three primary bond lengths irrespective of its coordination number; the grand mean of the primary Se^4+^—O^2−^ bond lengths is 1.705 Å and the observed range is 1.614–1.872 Å. There is a small increase in the shortest primary and mean primary Se^4+^—O^2−^ bond lengths with increasing coordination number.

#### 5.2.8. Se^6+^   

Se^6+^ occurs in only one coordination: [4] with a grand mean bond length of 1.636 Å for 187 coordination polyhedra. The shortest Se^6+^—O^2−^ distance is 1.578 Å in Cs_3_(HSeO_4_)_2_(H_2_PO_4_) (Troyanov *et al.*, 1998 ▸) with a bond valence of 1.731 v.u.; the O^2−^ involved does not bond to Cs^+^ but the H^+^ ions were not located and hence we cannot assess the incident bond-valence sum around the constituent O^2−^. However, there is an Se^6+^—O^2−^ distance of 1.582 Å in RbAu(SeO_4_)_2_ (Buechner & Wickleder, 2004 ▸) which involves a [1]-coordinated O^2−^ ion, and several other well refined structures have Se^6+^—O^2−^ distances of 1.583–1.590 Å, suggesting a lower bound of 1.580 Å on Se^6+^—O^2−^ distances. Examination of Fig. 7 ▸(*j*) shows that the bond-length distribution for Se^6+^—O^2−^ has a long tail to longer values. A value of 1.75 Å occurs in Cs_3_(HSeO_4_)_2_(H_2_PO_4_) (Troyanov *et al.*, 1998 ▸) which contains an acid selenate group. The constituent O^2−^ ion does not bond to P^5+^ or Cs^+^ and the resultant bond valence is 1.125 v.u., indicating that the constituent O^2−^ ion is an (OH)^−^ group and suggesting that the value of 1.75 Å is a valid distance. The next-shortest Se^6+^—O^2−^ distance is 1.73 Å in K_2_(HSeO_4_)_1.5_(H_2_PO_4_)_0.5_ (Jaouadi *et al.*, 2006 ▸); again this involves an acid selenate group with Se^6+^—O^2−^ and K^+^—O^2−^ bond valences of 1.183 and 0.149 = 1.332 v.u., again in accord with coordination to an H^+^ ion involved in strong hydrogen bonding. Thus Se^6+^—(OH)^−^ distances up to 1.75 Å seem reasonable.

### 5.3. Group 17 non-metals   

**Figure 8:**
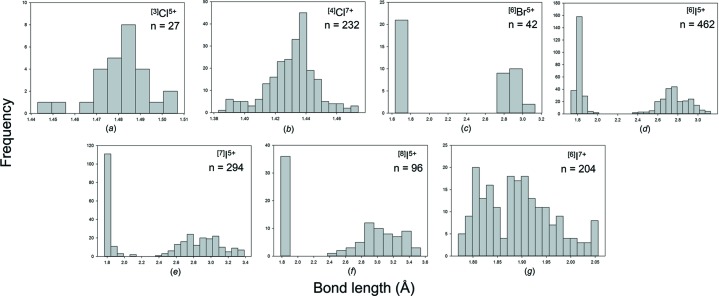
Bond-length distributions for selected configurations of the group 17 non-metal ions bonded to O^2−^: (*a*) ^[3]^Cl^5+^, (*b*) ^[4]^Cl^7+^, (*c*) ^[6]^Br^5+^, (*d*) ^[6]^I^5+^, (*e*) ^[7]^I^5+^, (*f*) ^[8]^I^5+^, (*g*) ^[6]^I^7+^.

We obtained a combined sample size of 1394 bonds and 248 coordination polyhedra for 14 configurations of the group 17 non-metals bonded to O^2−^. Table 3 ▸ gives the bond-length statistics for all configurations, and Fig. 8 ▸ shows the bond-length distribution for sample sizes deemed significant from the results of our sample size study (above). All bond-length and bond-valence distributions for group 17 non-metals bonded to O^2−^ are shown in Figs. S5 and S6.

#### 5.3.1. Br^5+^   

Br^5+^ occurs with three short distances in the range 1.63–1.70 Å and O^2−^—Br^5+^—O^2−^ angles in the range 99–108°, suggesting a stereoactive lone pair of electrons. Secondary bonds are observed on the side of the lone pair, and Br^5+^ has three coordination numbers: [6], [7] and [8] (Table 3 ▸) with grand mean bond lengths of 2.281, 2.578 and 2.671 Å, respectively. All coordinations are characterized by three short distances in the range 1.63–1.70 Å and two to four secondary bonds in the range 2.7–3.5 Å (Fig. 8 ▸ *c*).

#### 5.3.2. Br^7+^   

Br^7+^ occurs in one coordination: [4] with a grand mean bond length of 1.611 Å and a range of 1.603–1.623 Å for two polyhedra (Table 3 ▸).

#### 5.3.3. Cl^3+^   

The (ClO_2_)^−^ group has a grand mean bond length of 1.573 Å and a range of 1.557–1.592 Å, and is bent with O^2−^—Cl^3+^—O^2−^ angles in the range 108.4–111.4°. The fourth shortest interatomic distance lies in the range 2.90–3.65 Å for these structures. It is not clear where to draw the line as to what secondary bonds are significant; the shortest secondary bonds (2.901 Å ×2) occur in KClO_2_ (Smolentsev & Naumov, 2005 ▸).

#### 5.3.4. Cl^5+^   

Cl^5+^ is [3]-coordinated with a grand mean bond length of 1.481 Å and a range of 1.443–1.507 Å for nine coordination polyhedra. The O^2^—Cl^5+^—O^2−^ angles are in the range 103.3–107.4° and the coordination is trigonal prismatic with Cl^5+^ at the apical vertex. Next-nearest Cl^5+^—O^2−^ distances lie beyond 3.08 Å, over twice the mean bond length of the short bonds, and are not included as bonds.

#### 5.3.5. Cl^7+^   

Cl^7+^ is [4]-coordinated with a grand mean bond length of 1.431 Å, a range of 1.383–1.474 Å and a fairly symmetrical distribution for 65 coordination polyhedra.

#### 5.3.6. I^5+^   

I^5+^ shows a range of coordinations from [6] to [9], all of which show a very strong bimodal distribution of bond lengths [Figs. 8(*d*)–8(*f*)]. There are three short distances in the range 1.734–1.932 Å irrespective of coordination number, and three to six secondary bonds at much longer distances: ∼2.4–3.5 Å, suggestive of stereoactive lone-pair behaviour, with grand mean bond length increasing with coordination number: 2.294, 2.438, 2.587 and 2.699 Å, respectively (Table 3 ▸).

#### 5.3.7. I^7+^   

I^7+^ has two coordination numbers: [4] and [6], with a strong preference for [6] (Table 3 ▸). The grand mean bond lengths are 1.763 and 1.892 Å with ranges of 1.757–1.769 and 1.770–2.056 Å, respectively. The distribution of values for [6]-coordination (Fig. 8 ▸ *g*) appears somewhat bimodal, but we suggest that this is an artifact of the relatively small number of coordination polyhedra (34 coordination polyhedra).

## 6. Discussion   

### 6.1. Summary of bond-length dispersion analysis for ions with lone-pair electrons   

There are 14 cations with lone-pair electrons in our bond-length dispersion analysis: Cl^5+^, Cl^3+^, S^4+^, P^3+^, Br^5+^, Se^4+^, As^3+^, I^5+^, Te^4+^, Sb^3+^, Sn^2+^, Bi^3+^, Pb^2+^ and Tl^+^. Non-metals account for seven of these ions, metalloids for three, and poor metals for four. Although the metalloid and poor-metal ions are treated with their respective families in our series on bond-length distributions for ions bonded to O^2−^ (Gagné & Hawthorne, 2018 ▸), it is appropriate to briefly review the data for all ions here.

In their analysis of lone-pair stereoactive ions, Galy *et al.* (1975 ▸) give examples for which the lone-pair electrons are ‘fully stereoactive’, but state that in the majority of cases, they are observed in an ‘intermediate state’ between stereoactivity and inertness. This is what we observe in our data (see below); there is a minority of cases for which longer interatomic distances to the anions are 2–3× that of the mean bond length for the short bonds, leading to coordination numbers [2] to [4] and where the lone-pair electrons are arguably ‘fully stereoactive’. The rest of the data show longer interatomic distances that can be considered as bonded (secondary bonds), with a wide range of anisotropy.

For ions of period 3, Cl^5+^, S^4+^ and P^3+^ do not form secondary bonds, and occur with coordination numbers [2], [3] and [4]; the next-nearest anions occur at distances that are typically 2–3× that of the mean bond length for the short bonds. However, Cl^3+^ (exceptionally *ns* ^2^ *np* ^2^) is more ambiguous; in four of five structures, it is assigned a coordination number of [2]; in the other structure, KClO, there is the possibility of two longer bonds, where the nearest O^2−^ ions are at 1.565 Å ×2 (O—Cl—O angle 108.2°), 2.901 Å ×2 and 4.116 Å ×2. Although inclusion of the two distances at 2.901 Å is questionable, following the method of Gagné & Hawthorne (2016 ▸) resulted in their inclusion.

Period 4 ions, Br^5+^, Se^4+^ and As^3+^, generally show longer interatomic distances to O^2−^ on the side of the lone pair: in all cases for Br^5+^, in 189 of 202 coordination polyhedra for Se^4+^ (where the ion is otherwise clearly [3]- or [4]-coordinated), and in 15 of 28 coordination polyhedra for As^3+^. For polyhedra with coordination numbers [3] or [4], the next-nearest anions are usually observed at over twice the distance of the mean bond length for a coordination number of [3], *e.g.* in derriksite (Ginderow & Cesbron, 1983 ▸), Cu_4_(UO_2_)(SeO_3_)_2_(OH)_6_, Se—O are: 1.639, 1.695 (×2), 3.686 (×2), 3.736 (×2), 3.796 (×2) Å, *etc*. Of the 194 Se^4+^—O^2−^ coordination polyhedra we have collected with four bonds or more, the mean distance of the fourth bond is 2.94 Å; distances of 3.686 Å result in bond valences < 0.01 v.u., and can be disregarded as insignificant. The O—Se—O angles of 101.9° and 102.5° (×2) indicate stereoactivity of the lone pair.

Similar results were obtained for period 5 and 6 ions: in all cases, there are longer interatomic distances available to I^5+^ to form secondary bonds: 200 of 212 coordination polyhedra for Te^4+^, 33 of 54 coordination polyhedra for Sb^3+^, 23 of 50 coordination polyhedra for Sn^2+^, 201 of 231 coordination polyhedra for Bi^3+^, 254 of 276 coordination polyhedra for Pb^2+^, and for 68 of 74 coordination polyhedra for Tl^+^.

For data with CN > [4], *i.e.* for ions where the lone pair is not ‘fully stereoactive’ as defined by Galy *et al.* (1975 ▸), we are left with the following question: how do the bond-length distributions of the ions change as the arrangement varies from a stereoactive lone pair to an inert lone pair? Is the progression a function of anisotropy, *i.e.* is there an inverse relation between stereoactivity and coordination number, or can both intermediate and inert lone pairs be observed for any coordination number > [4]?

**Figure 9:**
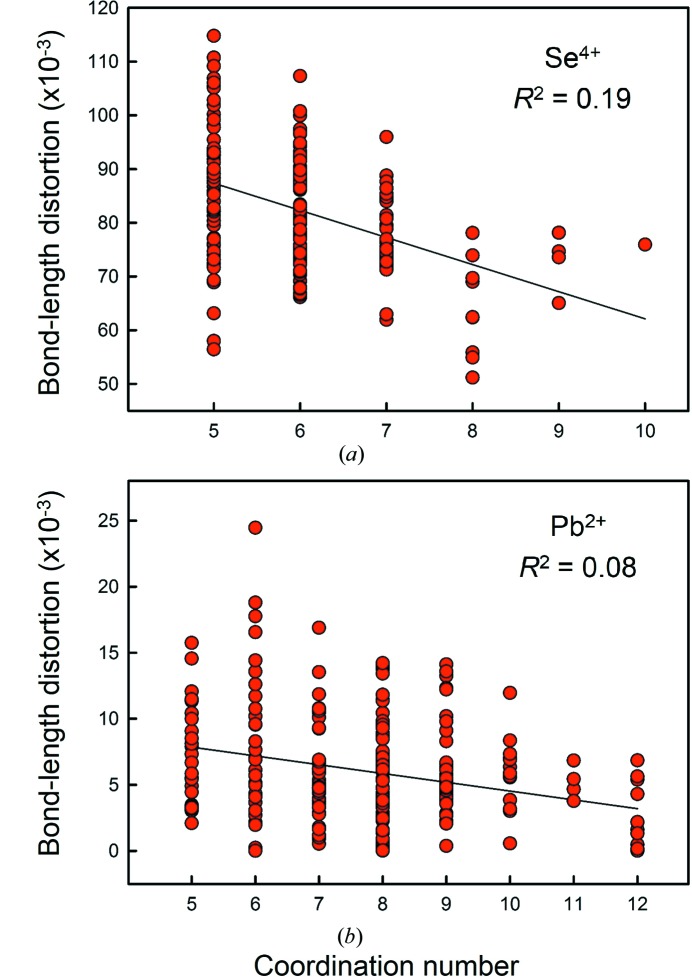
Bond-length distortion as a function of coordination number for (*a*) Se^4+^ and (*b*) Pb^2+^.

**Figure 10:**
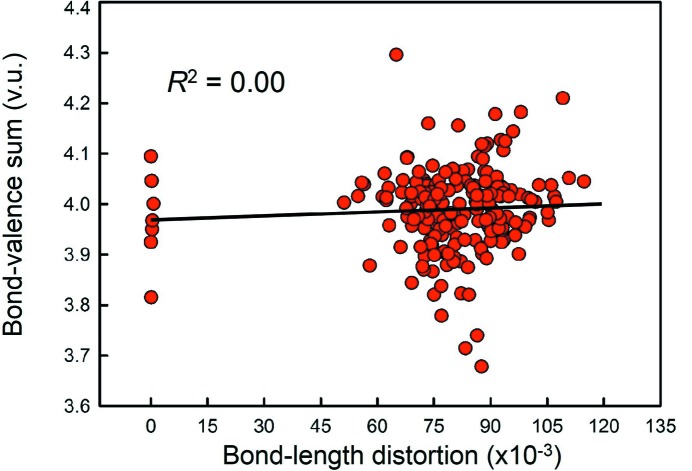
Correlation between lone-pair stereoactivity and bond-valence sum for Se^4+^ using the bond-valence parameters given by Gagné & Hawthorne (2015 ▸).

To resolve this issue, we look at Se^4+^ and Pb^2+^, and use the proposition of Brown (1988 ▸) that bond-length distortion is a measure of the stereoactivity of the lone pair. However, instead of the vector-based model used by Brown (1988 ▸), we use the scalar definition of bond-length distortion given by Brown & Shannon (1973 ▸), *i.e.* the root-mean-square deviation of the individual bond lengths from the mean value in the polyhedron. This definition is sufficient, as we are dealing with cations that exhibit the same kind of asymmetric distortion, *e.g.* Se^4+^ and Pb^2+^; if we were comparing lone pair and non-lone-pair cations, *e.g.* Pb^2+^ and ^[6]^Cu^2+^, we could not use scalar bond-length distortion as a possible indicator of lone-pair stereoactive behaviour because of the presence of very large centrosymmetric or pseudo-centrosymmetric distortion for non-lone-pair cations, *e.g.* ^[6]^Cu^2+^. Fig. 9 ▸ shows bond-length distortion as a function of coordination number for (*a*) Se^4+^ and (*b*) Pb^2+^. The correlations are significant at the 99% confidence level, with *p*-values of 2 × 10^−10^ and 3 × 10^−6^, respectively. However, we find that despite being significant, the inverse correlation between the degree of stereoactivity and coordination number is very weak, with *R* ^2^ = 0.19 and 0.08 for Se^4+^ and Pb^2+^, respectively. Although Se^4+^ is not observed with a ‘fully inert’ lone pair in our dataset, the results for Pb^2+^ show ions with intermediate to inert lone pairs for coordination numbers ≥ [6]. Therefore, we find no strong relation between lone-pair stereoactivity (as measured by bond-length anisotropy) and coordination number. Furthermore, analysis of bond-valence sums as a function of bond-length distortion shows no correlation between lone-pair stereoactivity and bond-valence sum incident at the cation for the bond-valence parameters of Gagné & Hawthorne (2015 ▸). This is shown in Fig. 10 ▸ for Se^4+^ (*R* ^2^ = 0.00, *p*-value = 0.36).

All data used here are deposited with the publications of the current series which deals with those ions; we encourage readers to carry out more detailed analysis.

### 6.2. Lone-pair stereoactivity in non-metals   

Here we take a closer look at lone-pair stereoactivity for non-metal ions bonded to O^2−^. There are two factors usually indicative of stereoactivity of lone-pair electrons: (1) a strongly anisotropic coordination environment, and (2) the presence of secondary bonds. As discussed above for period 3 non-metal ions, one does not lead to the other.

Let us take a closer look at the period 3 non-metal ions. These elements (P, S, Cl) all occur in more than one oxidation state, with 0 (*ns* ^0^ *np* ^0^), 1 (*ns* ^2^ *np* ^0^) or 2 (*ns* ^2^ *np* ^2^) lone-pair electrons: P^5+^ and P^3+^, S^6+^ and S^4+^, and Cl^7+^, Cl^5+^ and Cl^3+^_._ Because these ions occur in coordinations [2] to [4] and do not form secondary bonds (aside from the ambiguous case of Cl^3+^ in KClO described above), we may not infer stereoactivity for their lone-pair electrons *via* scalar bond-length distortion. Thus we examine the regularity of the bond angles to determine whether the lone pair(s) on these ions are stereoactive.

Bond angles in a tetrahedron are ideally 109.5°, and 〈O—*T*—O〉 angles in *T*O_4_ groups where *T* does not have a stereoactive lone pair of electrons are generally very close to 109.5°. As discussed by Gillespie & Nyholm (1957 ▸), O—*T*—O angles are less in the presence of lone-pair electrons due to the greater repulsive power of lone-pair electrons compared with bonded electrons. For these ions, we observe in our dataset O—*X*—O (*X* = P^3+^, S^4+^, Cl^5+^) angles of ∼ 97–100° for P^3+^, ∼99–107° for S^4+^ and ∼103–107° for Cl^5+^. In comparison, these angles are ∼99–108° for the three strong bonds of Br^5+^ structures that are lone-pair stereoactive. Thus we conclude that the lone-pair electrons of period 3 non-metal ions are stereoactive.

We also observe a marked difference in the preference of the non-metal elements for their *n* and *n*+2 oxidation states, between period 3 and periods 4 and 5. For period 3 ions, the numbers of coordination polyhedra observed for the *n* and *n*+2 (or *n*+4) oxidation states are seven *versus* 3650 for P^3+^ and P^5+^, 30 *versus* 890 for S^4+^ and S^6+^, and five, nine and 58 for Cl^3+^, Cl^5+^ and Cl^7+^. For periods 4 and 5 non-metal ions, the lower oxidation state is more common, *i.e.* with lone pair: thus the number of coordination polyhedra are 202 *versus* 187 for Se^4+^ and Se^6+^, nine *versus* two for Br^5+^ and Br^7+^, and 134 *versus* 36 for I^5+^ and I^7+^.

### 6.3. Polymerization of the PO_4_ group   

**Figure 11:**
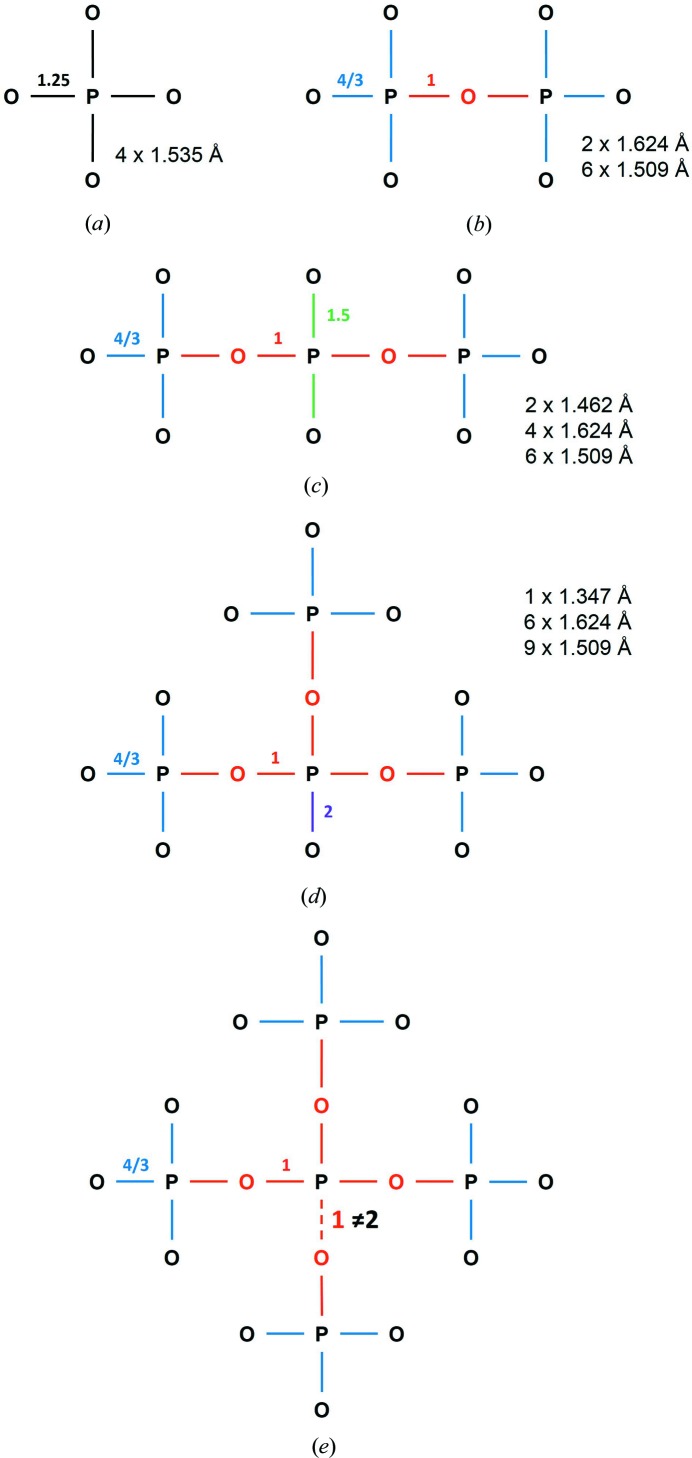
Polymerization of a PO_4_ group as a function of the number of bridging oxygen atoms (O_br_): (*a*) O_br_ = 0 (monomer), (*b*) O_br_ = 1 (dimer), (*c*) O_br_ = 2 (trimer), (*d*) O_br_ = 3, (*e*) O_br_ = 4. The valence-sum rule cannot be satisfied for the central P^5+^ ion in (*e*), and this unit does not exist.

The distribution of ^[4]^P^5+^—O^2−^ bond lengths (Fig. 7 ▸ *c*) shows several maxima and is significantly different from the distribution of ^[4]^P^5+^—O^2−^ bond lengths in minerals (Huminicki & Hawthorne, 2002 ▸) which shows only one maximum. Examination of the bond-valence distribution for ^[4]^P^5+^—O^2−^ (Fig. S4*e*) shows that the bond-valence distribution has its maximum at ∼1.25 v.u., as expected, but that other bond-valence distributions may be superimposed onto the main distribution to give it a multi-modal aspect. Polymerization of the PO_4_ group is a potential cause of this, so let us examine the polymerization of the PO_4_^3−^ ion from a bond-valence perspective. Geometrically, a PO_4_ group may link to up to four additional PO_4_ groups *via* bridging oxygen atoms (O_br_), as illustrated in Fig. 11 ▸ for high-symmetry environments. Fig. 11 ▸(*a*) shows an isolated PO_4_ group (a monomer). According to the bond-valence model (Brown, 2002 ▸, 2016 ▸), the electron density is split evenly into the four bonds of the group, giving a bond valence of 1.25 v.u. for each P—O bond. Using the bond-valence parameters of Gagné & Hawthorne (2015 ▸), this gives a predicted mean bond length of 1.535 Å. For a dimer (Fig. 11 ▸ *b*), with O_br_ = 1, the sum of the bond valences at O_br_ is equal to 2 v.u.; this gives 1 v.u. for each P—O_br_ bond, corresponding to a P—O distance of 1.624 Å. There are 4 v.u. left to distribute over the remaining three bonds for each PO_4_ group, giving a bond valence of 4/3 = 1.333 v.u. and a corresponding P—O distance of 1.509 Å. Thus the bonds of a dimer are ideally 2 × 1.624 Å and 6 × 1.509 Å. For a trimer (Fig. 11 ▸ *c*), with O_br_ = 2, a similar treatment leads to four P—O_br_ of 1 v.u. each, and six P—O bonds with 4/3 v.u. However, the PO_4_ group with two P—O_br_ bonds of 1 v.u. each is left with 3 v.u. to be distributed amongst its two other P—O bonds, that is 1.5 v.u. each (1.462 Å). Thus the bonds of a trimer are ideally 2 × 1.462 Å, 4 × 1.624 Å and 6 × 1.509 Å. For O_br_ = 3 (Fig. 11 ▸ *d*), the peripheral PO_4_ groups are as usual 3 × 4/3 and 1 × 1 v.u. The central PO_4_ group, however, has 3 × P—O_br_ bonds of 1 v.u. each, and the other P—O bond then adjusts to 2 v.u. This leaves the oxygen atom with no electron density to bond to other cations. Thus the bonds for O_br_ = 3 are ideally 1 × 1.347 Å, 6 × 1.624 Å and 9 × 1.509 Å. The last case is for a PO_4_ group which has four P—O_br_ bonds (Fig. 11 ▸ *e*). However, a bond-valence treatment shows that this arrangement is not possible: the four O_br_ ions are constrained to have 2 × 1 v.u. bonds, thus constraining the four P—O_br_ bonds to be 1.v.u. each for an incident bond-valence sum at the central P^5+^ of 4 v.u. The valence-sum rule is not satisfied for the central P^5+^ ions, and this unit cannot exist. In other words, the only non-bridging oxygen atom of the PO_4_ unit with three O_br_ (Fig. 11 ▸ *d*) has a bond valence of 2 v.u., and therefore cannot bond to another P atom (Fig. 11 ▸ *e*). The units of Fig. 11 ▸ may polymerize with each other in various combinations, but this will result in no ‘new’ bonding constraint.

We note that the cases discussed above are idealized; in practice, small deviations will occur for two reasons: (1) it is possible for the O_br_ ions to bond to other cations, thus lowering the bond-valence constraints of the P—O—P bonds to lower than 1 v.u., to which the rest of the bonds in the polymerized unit will adjust, *e.g.* Rb—O_br_ in RbWO(P_2_O_7_) (Mezaoui *et al.*, 2006 ▸); (2) the constraints given above are for structures of high symmetry for the PO_4_ tetrahedron. A lower symmetry (*i.e.* non-equivalent bonds) will result in slightly different bond-valence constraints, and hence observed bond lengths. These two cases result in a continuum of observed bond valences and bond lengths, with maxima in the distributions at the bond-valence constraints discussed above.

#### 6.3.1. Partitioning our PO_4_ dataset according to the number of bridging oxygen atoms   

**Figure 12:**
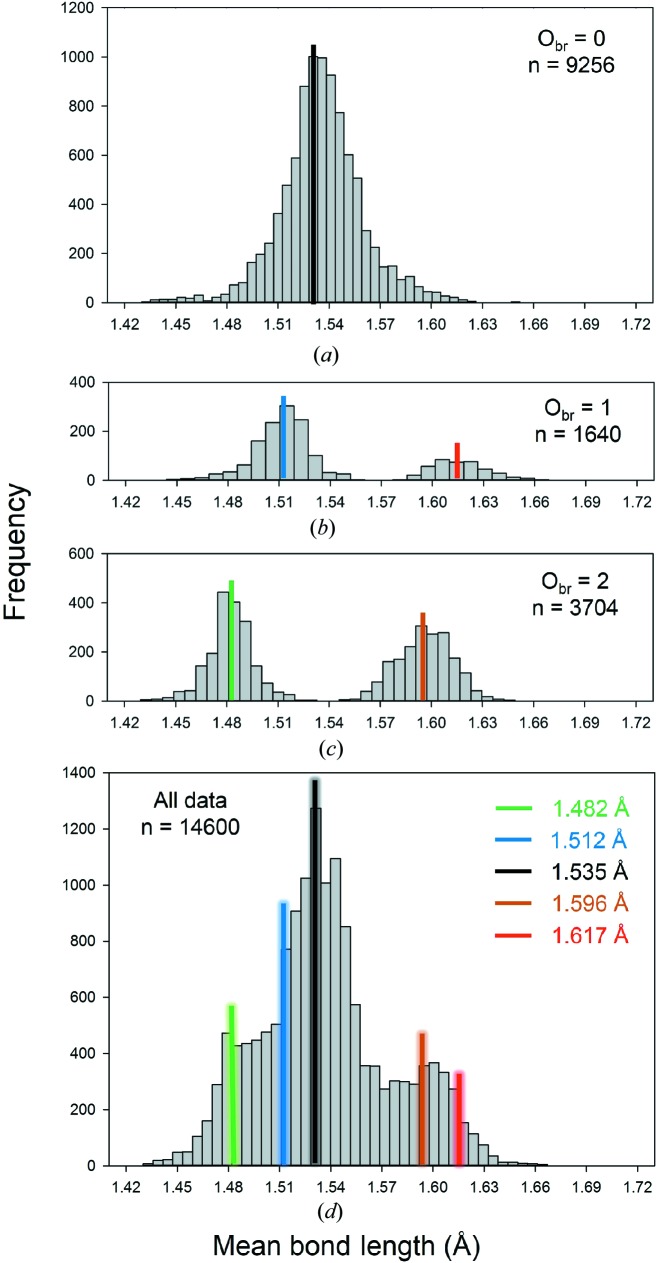
Bond-length distributions of PO_4_ tetrahedra for O_br_ = (*a*) 0, (*b*) 1 and (*c*) 2, with sample sizes of 9256, 1640 and 3704 bonds, respectively. The bond-length constraints are shown. The colour scheme of Fig. 11 ▸ is preserved, although bonds of 1 v.u for O_br_ = 1 and 2 give slightly different observed mean bond lengths, given in red (O_br_ = 1) and orange (O_br_ = 2).

Following the bond-valence requirements described above, we may split our PO_4_ dataset as a function of the number of O_br_ atoms. In Fig. 12 ▸, we give distributions for O_br_ = 0 (*a*), O_br_ = 1 (*b*) and O_br_ = 2 (*c*), with sample sizes of 2314, 410 and 926 coordination polyhedra, respectively. Of the 3650 coordination polyhedra in our P^5+^ dataset, there is none with O_br_ = 3 (Fig. 11 ▸ *d*).

Generally, our data shows a difference of over ∼0.06 Å between the second and third shortest P—O distances for PO_4_ tetrahedra with two O_br_ anions, and a difference of over ∼0.07 Å between the third and fourth shortest P—O distances for PO_4_ tetrahedra with one O_br_ anion.

Fig. 12 ▸(*a*) shows a regular distribution for O_br_ = 0, very similar to that obtained by Huminicki & Hawthorne (2002 ▸) from a review of phosphate minerals. They observed a mean bond length of 1.537 Å and bond lengths ranging from 1.43 to 1.64 Å; moreover, there were only two or three di- and poly phosphate minerals known at that time, and hence the data is almost completely for orthophosphate structures. We observe a mean bond length of 1.536 Å (expected from the bond-valence model: 1.535 Å) and a range of 1.43–1.65 Å. In Fig. 12 ▸(*b)*, for O_br_ = 1, we observe a mean bond length of 1.512 Å for the three shortest bonds and 1.617 Å for the longest bond (expected from the bond-valence model: 1.509 and 1.624 Å). In Fig. 12 ▸(*c*), for O_br_ = 2, we observe a mean bond length of 1.482 Å for the two shortest and 1.596 Å for the two longest bonds (expected from the bond-valence model: 1.462 and 1.624 Å). In Fig. 12 ▸(*d*), we show how Figs. 12(*a*)–12(*c*) combine to produce the observed parent distribution (Fig. 7 ▸ *c*). Moreover, the mean bond-valence sums for the structures with O_br_ = 0, 1 and 2 are identical in all cases, *i.e.* exactly 5.00 v.u.

There are more PO_4_ tetrahedra with O_br_ = 2 than O_br_ = 1; this is due to (1) the formation of PO_4_ chains of finite length, *e.g.* in Zn_5_(P_3_O_10_)_2_(H_2_O)_17_ (Averbuch-Pouchot *et al.*, 1975 ▸), (2) the formation of PO_4_ chains of infinite length, *e.g.* for NaNd(PO_3_)_4_ (Koizumi, 1976 ▸), (3) ring formation, *e.g.* for three PO_4_ tetrahedra in Ba_3_(P_3_O_9_)_2_(H_2_O)_6_ (Masse *et al.*, 1976 ▸), and (4) the formation of sheets with other strongly bonded cations, *e.g.* (NH_4_)_2_(SiP_4_O_13_) (Durif *et al.*, 1976 ▸) where PO_4_ polymerizes with other PO_4_ tetrahedra and SiO_6_ octahedra to form SiP_4_O_13_ sheets inter-linked by NH_4_ groups. Combinations of these arrangements also occur, *e.g.* in KTa(PO_3_)_2_(P_2_O_7_) (Nikolaev *et al.*, 1983 ▸) which combines infinite chains and dimers. PO_4_ tetrahedra with O_br_ = 1 may be relatively outnumbered by those with O_br_ = 2 in (1), and are absent in (2), (3) and (4), hence the relatively high abundance of PO_4_ tetrahedra with O_br_ = 2 observed in our dataset. A thorough discussion of the polymerization of PO_4_ groups goes beyond the scope of the present work, but synthesis efforts have evidently resulted in a remarkable diversity of these compounds.

### 6.4. Mean bond-length distributions   

**Figure 13:**
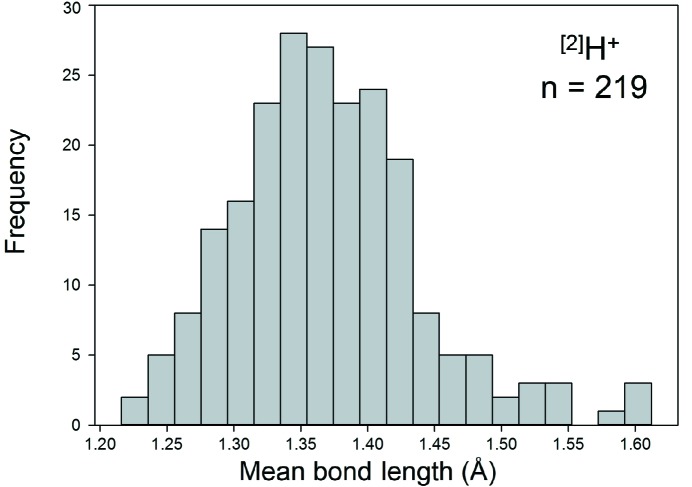
Mean bond-length distribution for ^[2]^H^+^ bonded to O^2−^.

The mean bond length distributions for hydrogen, the group 14–16 and group 17 non-metal ions bonded to O^2−^ are given in Figs. S7, S8 and S9, respectively. Those with adequate sample sizes (see sample size study above) are shown in Figs. 13 ▸, 14 ▸ and 15 ▸. Tables 4 ▸, 5 ▸ and 6 ▸ give the grand mean bond length and standard deviation, the minimum and maximum mean bond length (and range), the skewness and kurtosis of each distribution (where justified by sample size) and the number of coordination polyhedra and coordination numbers for all configurations observed.

As expected, we observe a narrow range of mean bond lengths for the strongly bonded non-metal ions (*e.g.* C^4+^, N^5+^, P^5+^, S^6+^), typically ∼0.03–0.10 Å, and a large range for those ions that display lone-pair stereoactivity (*e.g.* I^5+^, Se^4+^), typically ∼0.2 Å. This compares to a typical range in mean bond lengths of ∼0.20–0.25 Å for alkaline earth metal ions and ∼0.3–0.4 Å for alkali metal ions bonded to O^2−^ (Gagné & Hawthorne, 2016 ▸). The mean bond length distributions for the non-metal ions are typically Gaussian with no skew, in contrast to the alkali and alkaline earth metals which typically show a positive skew in their distributions.

#### 6.4.1. Bond-length distortion   

**Figure 16:**
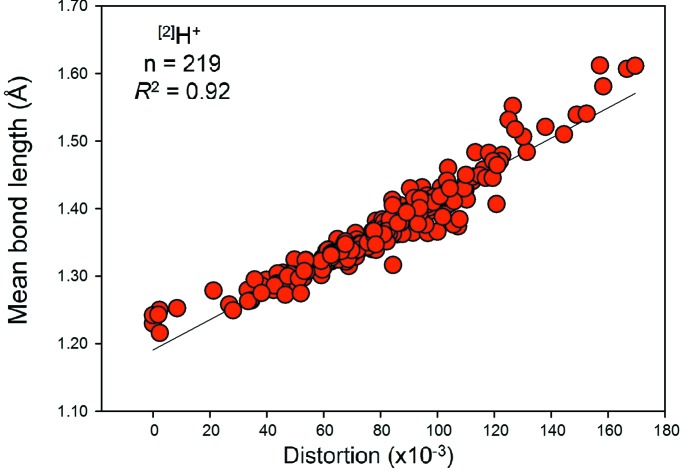
The effect of bond-length distortion on mean bond length for ^[2]^H^+^ bonded to O^2−^.

**Figure 17:**
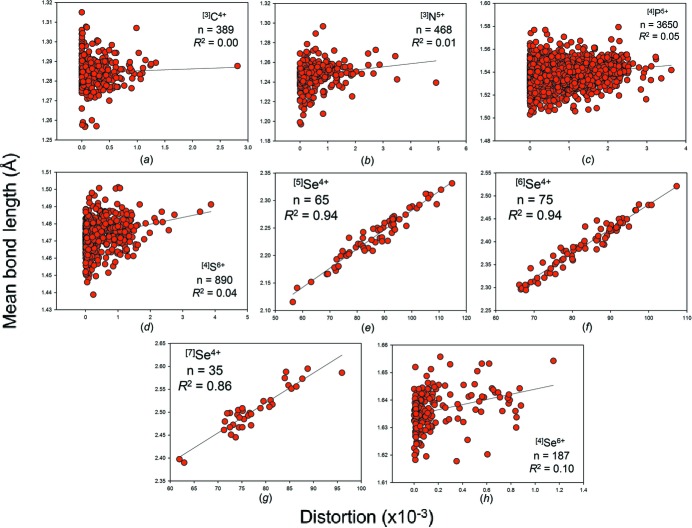
The effect of bond-length distortion on mean bond length for selected configurations of the group 14–16 non-metal ions bonded to O^2−^: (*a*) ^[3]^C^4+^, (*b*) ^[3]^N^5+^, (*c*) ^[4]^P^5+^, (*d*) ^[4]^S^6+^, (*e*) ^[5]^Se^4+^, (*f*) ^[6]^Se^4+^, (*g*) ^[7]^Se^4+^, (*h*) ^[4]^Se^6+^.

**Figure 18:**
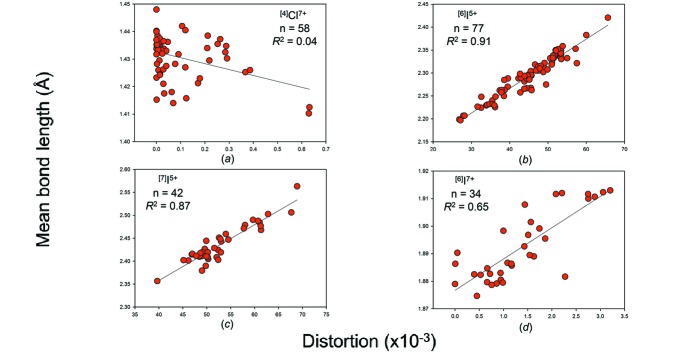
The effect of bond-length distortion on mean bond length for selected configurations of the group 17 non-metal ions bonded to O^2−^: (*a*) ^[4]^Cl^7+^, (*b*) ^[6]^I^5+^, (*c*) ^[7]^I^5+^, (*d*) ^[6]^I^7+^.

We give the bond-length distortion plots for hydrogen, the group 14–16 and group 17 non-metal ions in Figs. S10, S11 and S12, respectively, and in Figs. 16, 17 and 18 for those with adequate sample sizes. We use the definition of Brown & Shannon (1973 ▸) for distortion, *i.e.* the mean-square relative deviation of bond lengths from their average value. From these plots, we see that mean bond length correlates highly with bond-length distortion for ion configurations observed with distortion values > 10 × 10^−3^, *e.g.* *R* ^2^ = 0.92 for ^[2]^H^+^, 0.94 for ^[6]^Se^4+^, 0.91 for ^[6]^I^5+^, but very poorly for weakly distorted ion configurations (< 10 × 10^−3^).

#### 6.4.2. Factors affecting mean bond-length variations   

Gagné & Hawthorne (2017*b* ▸) gave a detailed examination of the potential factors affecting variation in mean bond length variation for 55 ion configurations, including ^[3]^C^4+^, ^[3]^N^5+^, ^[4]^P^5+^, ^[4]^S^6+^ and ^[4]^Se^6+^, but not any ion configurations with lone-pair electrons (due to their inadequate sample size). They concluded that, contrary to common usage, published correlations between mean bond length and mean coordination number of the bonded anions are not of general applicability to inorganic oxide and oxysalt structures. They (1) confirmed bond-length distortion as a causal factor of mean bond length variation and quantified its effect, and (2) found no correlation between mean bond length and the mean electronegativity and mean ionization energy of the next-nearest neighbours.

Let us examine the results for the non-metals ^[3]^C^4+^ (*n* = 67), ^[3]^N^5+^(*n* = 37), ^[4]^P^5+^ (*n* = 685), ^[4]^S^6+^ (*n* = 68) and ^[4]^Se^6+^ (*n* = 21). Student *t*-tests show that for (1) distortion, (2) mean coordination number of the bonded anions, (3) mean electronegativity and (4) mean ionization energy of the next-nearest neighbours, the only correlations significant at the 95% confidence level are bond-length distortion for ^[3]^C^4+^ (*p*-value = 0.030, *R* ^2^ = −0.07), ^[4]^P^5+^ (*p*-value = 8.5 × 10^−13^, *R* ^2^ = 0.01), ^[4]^S^6+^ (*p*-value = 1.2 × 10^−3^, *R* ^2^ = 0.15) and ^[4]^Se^6+^ (*p*-value = 4.8 × 10^−3^, *R* ^2^ = 0.35), and mean electronegativity (*p*-value = 9 × 10^−4^, *R* ^2^ = −0.01) and mean ionization energy (*p*-value = 1.1 × 10^−20^, *R* ^2^ = −0.07) of the next-nearest neighbours for P^5+^. A negative symbol before *R* ^2^ indicate that the observed correlation with mean bond length is negative.

As discussed by Gagné & Hawthorne (2017*b* ▸), values of *R* ^2^ and *p*-values vary significantly as a function of sample size, and analysis of ion configurations with less than ∼100 coordination polyhedra cannot be considered statistically reliable. In the above instances, however, the combination of a lack of statistical significance in most cases and low *R* ^2^ values for those cases that are statistically significant leads us to assume that mean bond length shows at most only little correlation with these factors in oxyanions. For ^[4]^P^5+^, the very large sample size of 685 coordination polyhedra ensures that any regression analysis should be independent of sample-size effects; in this case, mean bond length has near-negligible correlations with bond-length distortion, and mean electronegativity and mean ionization energy of the next-nearest neighbours.

It is apparent from Figs. 16 ▸ ▸–18 ▸ that bond-length distortion is the most important cause of mean bond-length variation for ions with stereoactive lone-pair electrons. On the other hand, highly distorted ion configurations are unusual, and distortion may only account for a very limited range of mean bond-length variation for typical ion configurations. This led Gagné & Hawthorne (2017*b* ▸) to assign the wide variation in mean bond length for individual ion configurations as a result of the inability of crystal structures to attain their ideal (*a priori*) bond lengths within the constraints of space-group symmetry. This is certainly the case for strongly bonded cations, *e.g.* oxyanions, for which the stress produced by the inability of a structure to follow its *a priori* bond lengths seems the most probable cause of mean bond length variation.

## 7. Summary   

(1) We have examined the bond-length distributions for three configurations of the H^+^ ion, 16 configurations of the group 14–16 non-metal ions, and seven configurations of the group 17 ions bonded to O^2−^, for 223 coordination polyhedra and 452 bond lengths for the H^+^ ion, 5826 coordination polyhedra and 22 784 bond lengths for the group 14–16 non-metal ions, and 248 coordination polyhedra and 1394 bond lengths for the group 17 non-metal ions.

(2) H⋯O and O—H + H⋯O distances correlate with O⋯O distance, with *R* ^2^ = 0.94 and 0.96, respectively. The following equations may be used to more accurately locate the hydrogen atom in a structure refined by X-ray diffraction: (1) H⋯O = 1.273 × O⋯O − 1.717 Å; (2) O—H + H⋯O = 1.068 × O⋯O − 0.170 Å.

(3) We find that for non-metal ions that occur with lone-pair electrons bonded to O^2−^, the most observed state out of the *n* *versus* *n*+2 oxidation state is that of higher oxidation state for period 3 cations, and lower oxidation state for period 4 and 5 cations.

(4) Observed O—*X*—O bond angles indicate that the period 3 non-metal ions P^3+^, S^4+^, Cl^3+^ and Cl^5+^ are lone-pair stereoactive when bonded to O^2−^, even though they do not form secondary bonds.

(5) We find no strong correlation between lone-pair stereoactivity and coordination number when including secondary bonds, whereby both intermediate and inert lone pairs may occur for any coordination number > [4] for the same cation. We also find no correlation between lone-pair stereoactivity and bond-valence sum at the central cation; this finding is in accord with the valence-sum rule.

(6) We show that in synthetic compounds, PO_4_ polymerizes with *via* one or two bridging oxygen atoms, but not by three. Partitioning our PO_4_ dataset shows that multi-modality in the distribution of bond lengths is caused by the different bond-valence constraints that arise for O_br_ = 0, 1 and 2.

(7) We observe variations in mean bond lengths of ∼0.03–0.10 Å for strongly bonded oxyanions of non-metal cations, and ∼0.2 Å for non-metal ions that display lone-pair stereoactivity.

(8) For strongly bonded cations, *e.g.* oxyanions, the most probable cause of mean bond-length variation is the effect of structure type, *i.e.* stress produced by the inability of a structure to adopt its *a priori* bond lengths. For ions with stereoactive lone-pair electrons, the most probable cause of variation is bond-length distortion.

## Supplementary Material

Figs. S1 to S12. DOI: 10.1107/S2052520617017541/bm5097sup1.pdf

Raw data file for Br5+. DOI: 10.1107/S2052520617017541/bm5097sup2.txt

Raw data file for Br7+. DOI: 10.1107/S2052520617017541/bm5097sup3.txt

Raw data file for C4+. DOI: 10.1107/S2052520617017541/bm5097sup4.txt

Raw data file for Cl3+. DOI: 10.1107/S2052520617017541/bm5097sup5.txt

Raw data file for Cl5+. DOI: 10.1107/S2052520617017541/bm5097sup6.txt

Raw data file for Cl7+. DOI: 10.1107/S2052520617017541/bm5097sup7.txt

Raw data file for H+. DOI: 10.1107/S2052520617017541/bm5097sup8.txt

Raw data file for I5+. DOI: 10.1107/S2052520617017541/bm5097sup9.txt

Raw data file for I7+. DOI: 10.1107/S2052520617017541/bm5097sup10.txt

Raw data file for N5+. DOI: 10.1107/S2052520617017541/bm5097sup11.txt

Raw data file for P3+. DOI: 10.1107/S2052520617017541/bm5097sup12.txt

Raw data file for P5+. DOI: 10.1107/S2052520617017541/bm5097sup13.txt

Raw data file for S4+. DOI: 10.1107/S2052520617017541/bm5097sup14.txt

Raw data file for S6+. DOI: 10.1107/S2052520617017541/bm5097sup15.txt

Raw data file for Se4+. DOI: 10.1107/S2052520617017541/bm5097sup16.txt

Raw data file for Se6+. DOI: 10.1107/S2052520617017541/bm5097sup17.txt

## Figures and Tables

**Figure 14:**
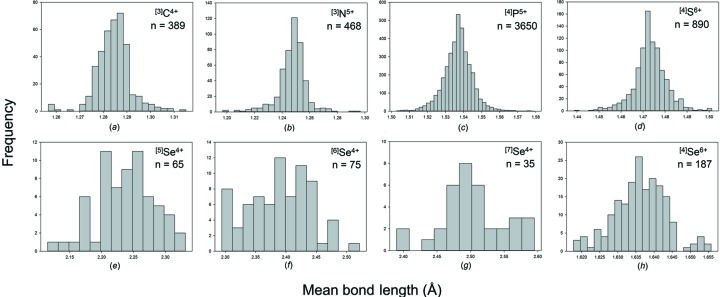
Mean bond-length distributions for selected configurations of the group 14–16 non-metal ions bonded to O^2−^: (*a*) ^[3]^C^4+^, (*b*) ^[3]^N^5+^, (*c*) ^[4]^P^5+^, (*d*) ^[4]^S^6+^, (*e*) ^[5]^Se^4+^, (*f*) ^[6]^Se^4+^, (*g*) ^[7]^Se^4+^, (*h*) ^[4]^Se^6+^.

**Figure 15:**
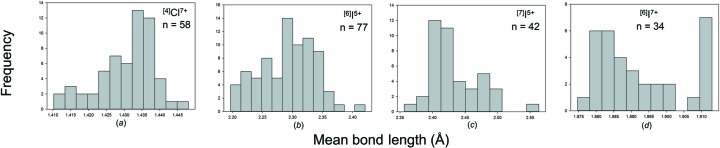
Mean bond-length distributions for selected configurations of the group 17 non-metal ions bonded to O^2−^: (*a*) ^[4]^Cl^7+^, (*b*) ^[6]^I^5+^, (*c*) ^[7]^I^5+^, (*d*) ^[6]^I^7+^.

**Table 1:** Bond-length statistics for the hydrogen ion bonded to O^2−^

Ion	Coordination number	Number of bonds	Number of coordination polyhedra	Mean bond length (Å)	Standard deviation (Å)	Range (Å)	Maximum bond length (Å)	Minimum bond length (Å)	Skewness	Kurtosis
H^+^	2	438	219	1.370	0.402	1.357	2.275	0.918	0.2	−1.6
	3	6	2	1.916	0.692	1.550	2.490	0.940	–	–
	4	8	2	2.233	0.746	1.723	2.663	0.940	–	–

**Table 2:** Bond-length statistics for the group 14–16 non-metal ions bonded to O^2−^

Ion	Coordination number	Number of bonds	Number of coordination polyhedra	Mean bond length (Å)	Standard deviation (Å)	Range (Å)	Maximum bond length (Å)	Minimum bond length (Å)	Skewness	Kurtosis
C^4+^	3	1167	389	1.284	0.020	0.156	1.384	1.228	0.1	1.1
N^5+^	3	1404	468	1.247	0.029	0.221	1.371	1.150	0.0	0.4
	4	12	3	1.385	0.005	0.020	1.397	1.377	–	–
P^3+^	3	21	7	1.536	0.043	0.220	1.675	1.455	–	–
P^5+^	4	14600	3650	1.537	0.039	0.266	1.696	1.430	0.3	-0.2
S^4+^	3	90	30	1.529	0.015	0.088	1.574	1.486	–	–
S^6+^	4	3560	890	1.473	0.027	0.260	1.652	1.392	1.0	2.8
Se^4+^	3	24	8	1.691	0.029	0.113	1.752	1.639	–	–
	4	20	5	2.027	0.570	1.565	3.213	1.648	–	–
	5	325	65	2.237	0.664	1.800	3.425	1.625	0.5	−1.7
	6	450	75	2.390	0.692	1.771	3.394	1.623	0.1	−1.9
	7	245	35	2.503	0.699	1.925	3.539	1.614	−0.2	−1.9
	8	72	9	2.530	0.646	1.707	3.364	1.657	−0.4	−1.7
	9	36	4	2.728	0.740	1.896	3.570	1.674	–	–
	10	10	1	2.882	0.794	1.867	3.565	1.698	–	–
Se^6+^	4	748	187	1.636	0.023	0.149	1.727	1.578	1.3	3.1

**Table 3:** Bond-length statistics for the group 17 non-metal ions bonded to O^2−^

Ion	Coordination number	Number of bonds	Number of coordination polyhedra	Mean bond length (Å)	Standard deviation (Å)	Range (Å)	Maximum bond length (Å)	Minimum bond length (Å)	Skewness	Kurtosis
Cl^3+^	2	8	4	1.573	0.011	0.035	1.592	1.557	–	–
	4	4	1	2.233	0.668	1.336	2.901	1.565	–	–
Cl^5+^	3	27	9	1.481	0.013	0.064	1.507	1.443	−1.0	2.1
Cl^7+^	4	232	58	1.431	0.016	0.091	1.474	1.383	-0.4	0.7
Br^5+^	6	42	7	2.281	0.629	1.496	3.131	1.635	0.0	−2.0
	7	7	1	2.578	0.806	1.727	3.361	1.634	–	–
	8	8	1	2.671	0.805	1.897	3.552	1.655	–	–
Br^7+^	4	8	2	1.611	0.007	0.020	1.623	1.603	–	–
I^5+^	6	450	75	2.294	0.492	1.392	3.126	1.734	0.1	−1.8
	7	273	39	2.438	0.563	1.614	3.385	1.771	0.0	−1.7
	8	96	12	2.587	0.632	1.756	3.542	1.786	−0.3	−1.7
	9	27	3	2.699	0.654	1.803	3.586	1.783	–	–
I^7+^	4	8	2	1.763	0.004	0.012	1.769	1.757	–	–
	6	204	34	1.892	0.071	0.286	2.056	1.770	0.4	−0.5

**Table 4:** Mean bond-length statistics for the hydrogen ion bonded to O^2−^

Ion	Coordination number	Number of coordination polyhedra	Grand mean bond length (Å)	Standard deviation (Å)	Mean bond length range (Å)	Maximum mean bond length (Å)	Minimum mean bond length (Å)	Skewness	Kurtosis
H^+^	2	219	1.370	0.071	0.396	1.612	1.216	0.8	1.2
	3	2	1.916	0.047	0.093	1.962	1.869	–	–
	4	2	2.232	0.000	0.001	2.233	2.232	–	–

**Table 5:** Mean bond-length statistics for the group 14–16 non-metal ions bonded to O^2−^

Ion	Coordination number	Number of coordination polyhedra	Grand mean bond length (Å)	Standard deviation (Å)	Mean bond length range (Å)	Maximum mean bond length (Å)	Minimum mean bond length (Å)	Skewness	Kurtosis
C^4+^	3	389	1.284	0.007	0.058	1.315	1.257	−0.1	3.2
N^5+^	3	468	1.247	0.010	0.100	1.297	1.197	−0.7	4.8
	4	3	1.385	0.004	0.009	1.390	1.381	–	–
P^3+^	3	7	1.656	0.011	0.034	1.675	1.641	–	–
P^5+^	4	3650	1.537	0.008	0.076	1.579	1.503	0.0	2.0
S^4+^	3	30	1.529	0.009	0.035	1.543	1.508	–	–
S^6+^	4	890	1.473	0.007	0.063	1.501	1.439	−0.1	2.1
Se^4+^	3	8	1.691	0.010	0.032	1.709	1.676	–	–
	4	5	2.027	0.051	0.108	2.089	1.982	–	–
	5	65	2.237	0.045	0.216	2.331	2.115	−0.2	0.0
	6	75	2.390	0.052	0.227	2.521	2.294	0.0	−0.6
	7	35	2.503	0.049	0.205	2.595	2.390	–	–
	8	9	2.530	0.068	0.205	2.642	2.437	–	–
	9	4	2.728	0.071	0.173	2.805	2.632	–	–
	10	1	2.882	–	0.000	2.882	2.882	–	–
Se^6+^	4	187	1.636	0.007	0.038	1.656	1.618	0.0	0.3

**Table 6:** Mean bond length statistics for the group 17 non-metal ions bonded to O^2−^

Ion	Coordination number	Number of coordination polyhedra	Grand mean bond length (Å)	Standard deviation (Å)	Mean bond length range (Å)	Maximum mean bond length (Å)	Minimum mean bond length (Å)	Skewness	Kurtosis
Cl^3+^	2	4	1.573	0.011	0.022	1.586	1.564	–	–
	4	1	2.233	–	0.000	2.233	2.233	–	–
Cl^5+^	3	9	1.483	0.006	0.014	1.490	1.476	–	–
Cl^7+^	4	58	1.431	0.008	0.038	1.448	1.410	–	–
Br^5+^	6	7	2.281	0.040	0.103	2.345	2.242	–	–
	7	1	2.578	–	0.000	2.578	2.578	–	–
	8	1	2.671	–	0.000	2.671	2.671	–	–
Br^7+^	4	2	1.611	0.005	0.007	1.614	1.608	–	–
I^5+^	6	75	2.294	0.045	0.224	2.421	2.197	−0.1	−0.1
	7	39	2.438	0.039	0.207	2.563	2.357	1.0	1.5
	8	12	2.587	0.045	0.175	2.687	2.512	–	–
	9	3	2.699	0.006	0.012	2.704	2.692	–	–
I^7+^	4	2	1.763	0.003	0.004	1.765	1.761	–	–
	6	34	1.892	0.012	0.038	1.913	1.875	–	–

## Footnotes

1 There are notable exceptions to this, *e.g.* the structures of PbO, Na_4_SnO_3_ (Brown, 2011 ▸) and Sr_3_Te_4_O_11_ (Christy & Mills, 2013 ▸)].
