# Supplementary material for: Bond-length distributions for ions bonded to oxygen: results for the non-metals and discussion of lone-pair stereoactivity and the polymerization of PO4
Source: Acta Crystallogr B Struct Sci Cryst Eng Mater. 2018 Jan 13;74(Pt 1):79–96. doi: 10.1107/S2052520617017541 (PMC5798399; doi:10.1107/S2052520617017541)
Supplement: Supplementary file 1 [file b-74-00079-sup1.pdf]

STRUCTURAL SCIENCE
CRYSTAL ENGINEERING
MATERIALS

Volume 74 (2018)

Supporting information for article:

Bond-length distributions for ions bonded to oxygen: results for the non-metals and discussion of lone-pair stereoactivity and the polymerization of PO4

Olivier Charles Gagné and Frank Christopher Hawthorne

Figure S1 Bond-length distributions for all configurations of the hydrogen ion bonded to O^{2-}: (*a*) $^{[2]}H^+$, (*b*) $^{[3]}H^+$, (*c*) $^{[4]}H^+$.

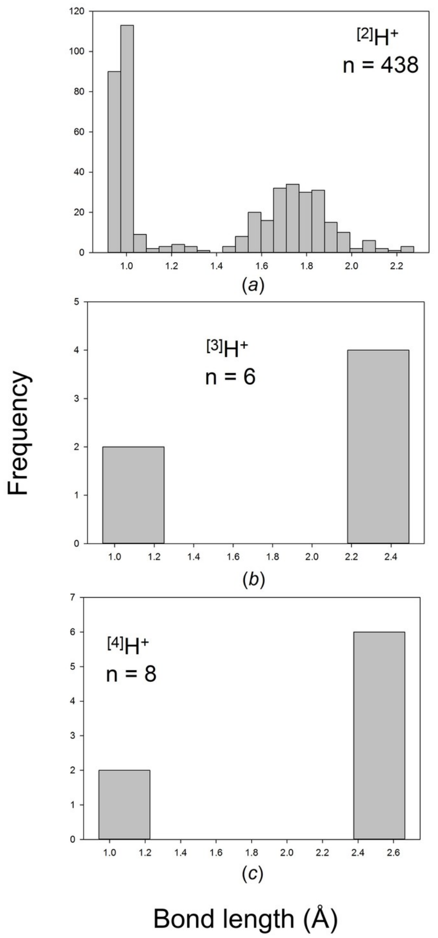

[2]H+
n = 438
(a)
[3]H+
n = 6
(b)
[4]H+
n = 8
(c)
Frequency
Bond length (Å)

Figure S2 Bond-valence distributions for all configurations of the hydrogen ion bonded to O^{2-}: (*a*) $^{[2]}H^{+}$, (*b*) $^{[3]}H^{+}$, (*c*) $^{[4]}H^{+}$.

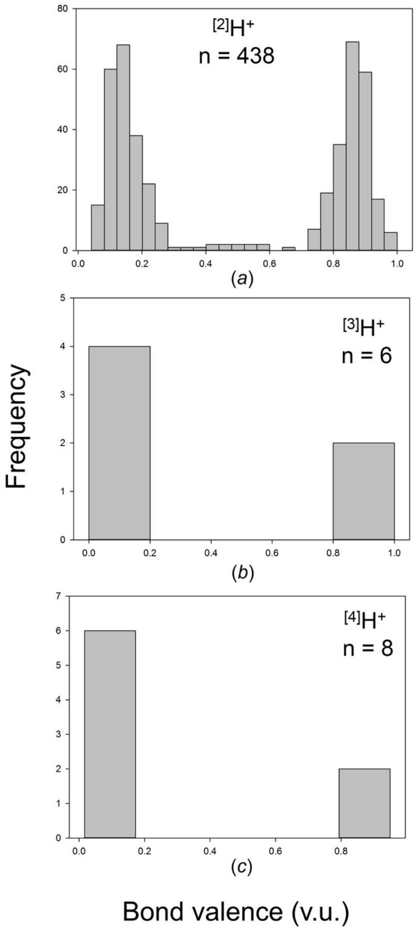
[2]H+
n = 438
(a)
[3]H+
n = 6
(b)
[4]H+
n = 8
(c)
Frequency
Bond valence (v.u.)

Figure S3 Bond-length distributions for all configurations of the group 14-16 non-metal ions bonded to O^{2-}: (*a*) $^{[3]}C^{4+}$, (*b*) $^{[3]}N^{5+}$, (*c*) $^{[4]}N^{5+}$, (*d*) $^{[3]}P^{3+}$, (*e*) $^{[4]}P^{5+}$, (*f*) $^{[3]}S^{4+}$, (*g*) $^{[4]}S^{6+}$, (*h*) $^{[3]}Se^{4+}$, (*i*) $^{[4]}Se^{4+}$, (*j*) $^{[5]}Se^{4+}$, (*k*) $^{[6]}Se^{4+}$, (*l*) $^{[7]}Se^{4+}$, (*m*) $^{[8]}Se^{4+}$, (*n*) $^{[9]}Se^{4+}$, (*o*) $^{[10]}Se^{4+}$, (*p*) $^{[4]}Se^{6+}$.

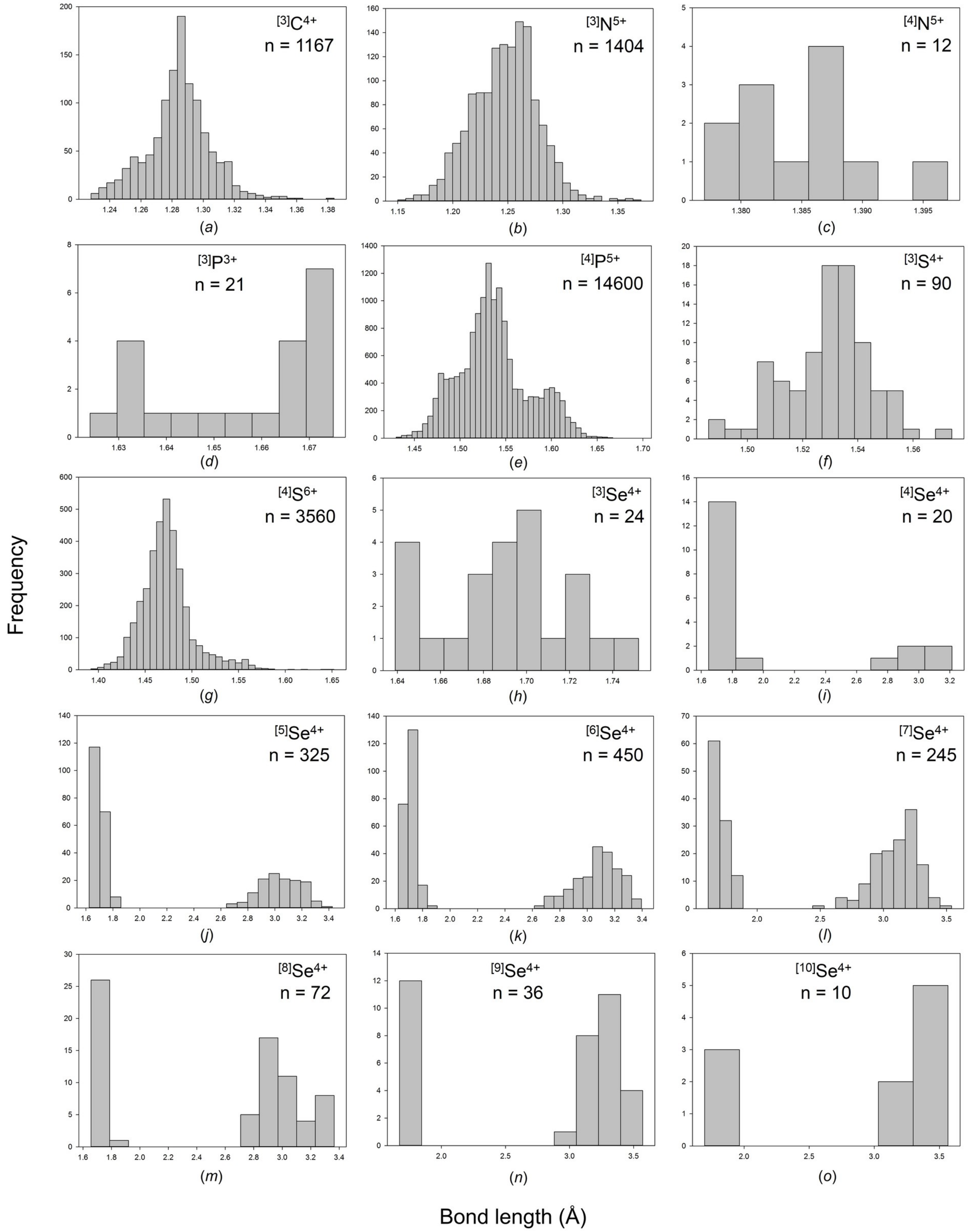
[3]C4+
n = 1167
(a)
[3]N5+
n = 1404
(b)
[4]N5+
n = 12
(c)
[3]P3+
n = 21
(d)
[4]P5+
n = 14600
(e)
[3]S4+
n = 90
(f)
[4]S6+
n = 3560
(g)
[3]Se4+
n = 24
(h)
[4]Se4+
n = 20
(i)
[5]Se4+
n = 325
(j)
[6]Se4+
n = 450
(k)
[7]Se4+
n = 245
(l)
[8]Se4+
n = 72
(m)
[9]Se4+
n = 36
(n)
[10]Se4+
n = 10
(o)
Frequency
Bond length (Å)

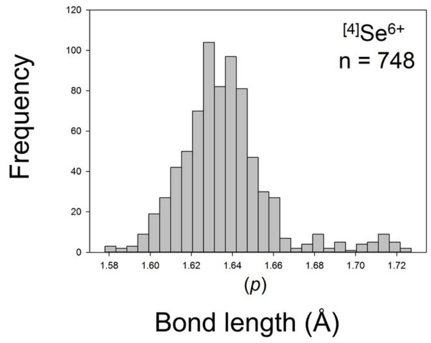

Frequency
120
100
80
60
40
20
0
1.58
1.60
1.62
1.64
1.66
1.68
1.70
1.72
[4]Se6+
n = 748
(p)
Bond length (Å)

Figure S4 Bond-valence distributions for all configurations of the group 14-16 non-metal ions bonded to O^{2-}: (*a*) $^{[3]}C^{4+}$, (*b*) $^{[3]}N^{5+}$, (*c*) $^{[4]}N^{5+}$, (*d*) $^{[3]}P^{3+}$, (*e*) $^{[4]}P^{5+}$, (*f*) $^{[3]}S^{4+}$, (*g*) $^{[4]}S^{6+}$, (*h*) $^{[3]}Se^{4+}$, (*i*) $^{[4]}Se^{4+}$, (*j*) $^{[5]}Se^{4+}$, (*k*) $^{[6]}Se^{4+}$, (*l*) $^{[7]}Se^{4+}$, (*m*) $^{[8]}Se^{4+}$, (*n*) $^{[9]}Se^{4+}$, (*o*) $^{[10]}Se^{4+}$, (*p*) $^{[4]}Se^{6+}$.

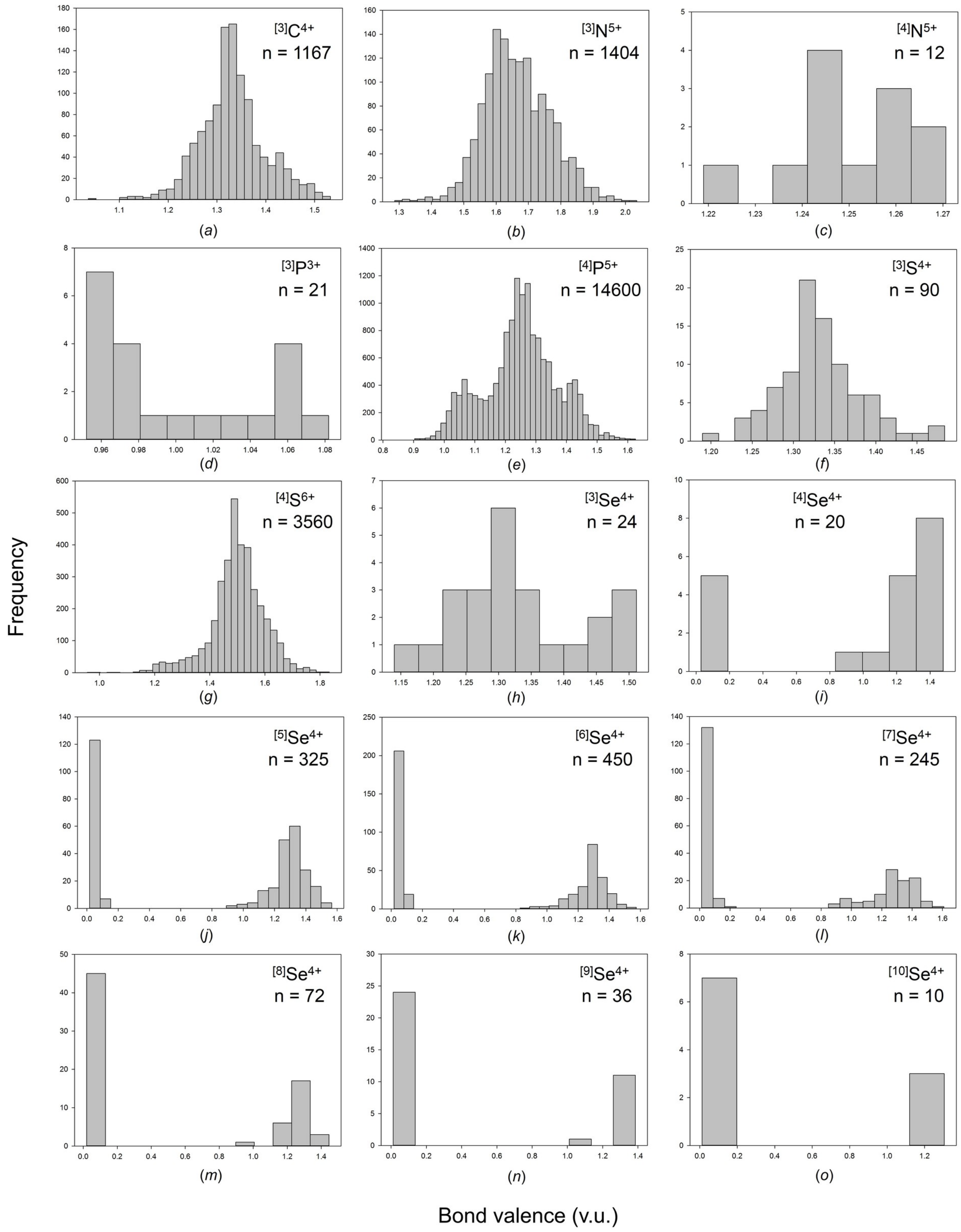
[3]C4+
n = 1167
(a)
[3]N5+
n = 1404
(b)
[4]N5+
n = 12
(c)
[3]P3+
n = 21
(d)
[4]P5+
n = 14600
(e)
[3]S4+
n = 90
(f)
[4]S6+
n = 3560
(g)
[3]Se4+
n = 24
(h)
[4]Se4+
n = 20
(i)
[5]Se4+
n = 325
(j)
[6]Se4+
n = 450
(k)
[7]Se4+
n = 245
(l)
[8]Se4+
n = 72
(m)
[9]Se4+
n = 36
(n)
[10]Se4+
n = 10
(o)
Frequency
Bond valence (v.u.)

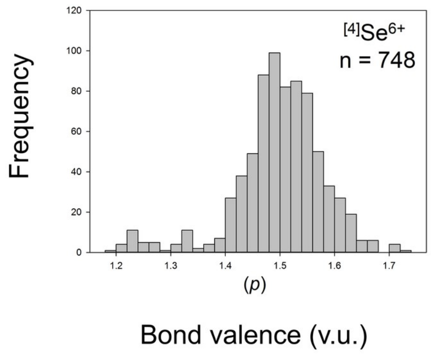
[4]Se6+
n = 748
Frequency
120
100
80
60
40
20
0
1.2
1.3
1.4
1.5
1.6
1.7
(p)
Bond valence (v.u.)

Figure S5 Bond-length distributions for all configurations of the group 17 non-metal ions bonded to O^{2-}: (*a*) $^{[2]}Cl^{3+}$, (*b*) $^{[4]}Cl^{3+}$, (*c*) $^{[3]}Cl^{5+}$, (*d*) $^{[4]}Cl^{7+}$, (*e*) $^{[6]}Br^{5+}$, (*f*) $^{[7]}Br^{5+}$, (*g*) $^{[8]}Br^{5+}$, (*h*) $^{[4]}Br^{7+}$, (*i*) $^{[6]}I^{5+}$, (*j*) $^{[7]}I^{5+}$, (*k*) $^{[8]}I^{5+}$, (*l*) $^{[9]}I^{5+}$, (*m*) $^{[4]}I^{7+}$, (*n*) $^{[6]}I^{7+}$.

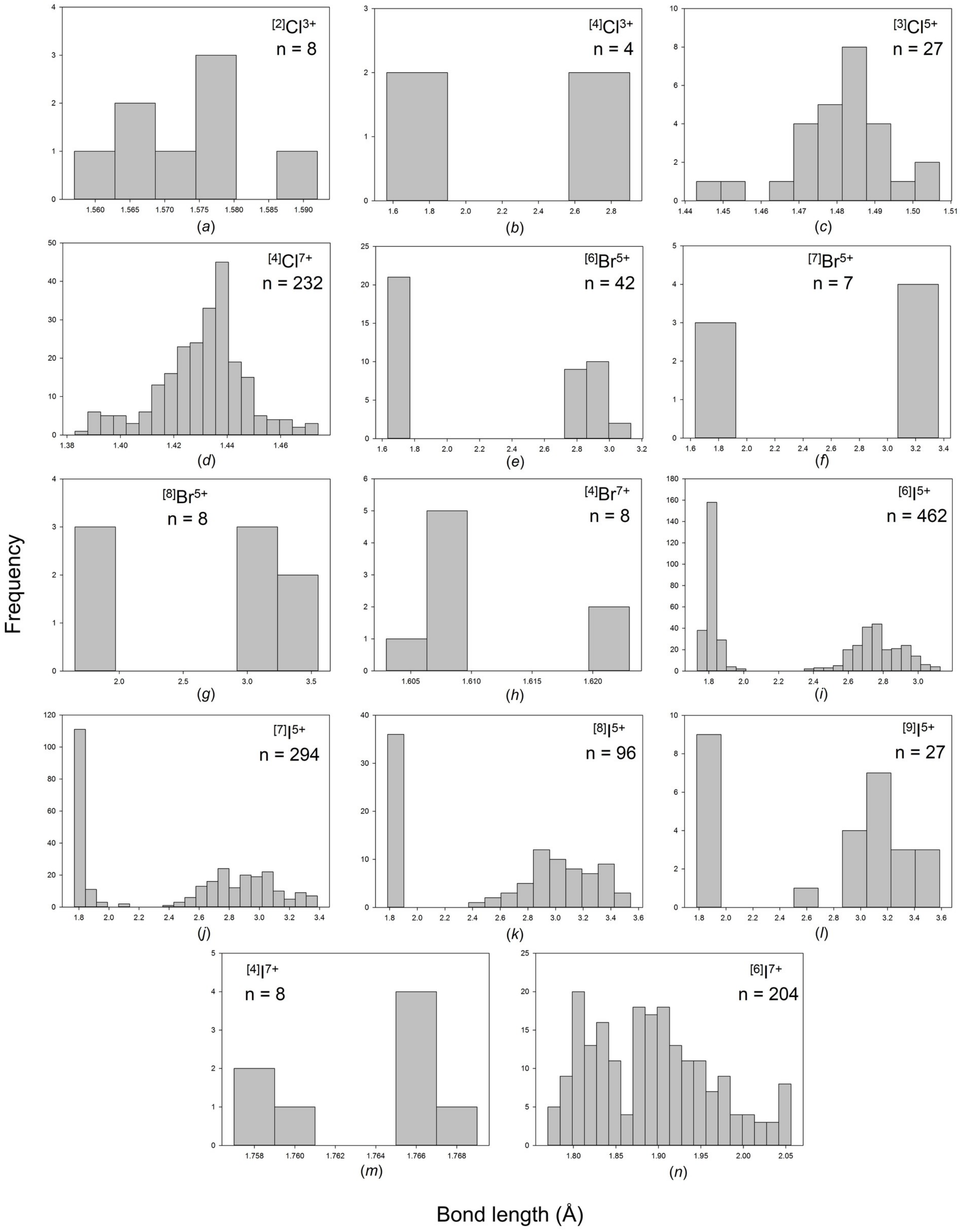
[2]Cl3+
n = 8
(a)
[4]Cl3+
n = 4
(b)
[3]Cl5+
n = 27
(c)
[4]Cl7+
n = 232
(d)
[6]Br5+
n = 42
(e)
[7]Br5+
n = 7
(f)
[8]Br5+
n = 8
(g)
[4]Br7+
n = 8
(h)
[6]I5+
n = 462
(i)
[7]I5+
n = 294
(j)
[8]I5+
n = 96
(k)
[9]I5+
n = 27
(l)
[4]I7+
n = 8
(m)
[6]I7+
n = 204
(n)
Frequency
Bond length (Å)

Figure S6 Bond-valence distributions for all configurations of the group 17 non-metal ions bonded to O^{2-}: (*a*) $^{[2]}Cl^{3+}$, (*b*) $^{[4]}Cl^{3+}$, (*c*) $^{[3]}Cl^{5+}$, (*d*) $^{[4]}Cl^{7+}$, (*e*) $^{[6]}Br^{5+}$, (*f*) $^{[7]}Br^{5+}$, (*g*) $^{[8]}Br^{5+}$, (*h*) $^{[4]}Br^{7+}$, (*i*) $^{[6]}I^{5+}$, (*j*) $^{[7]}I^{5+}$, (*k*) $^{[8]}I^{5+}$, (*l*) $^{[9]}I^{5+}$, (*m*) $^{[4]}I^{7+}$, (*n*) $^{[6]}I^{7+}$.

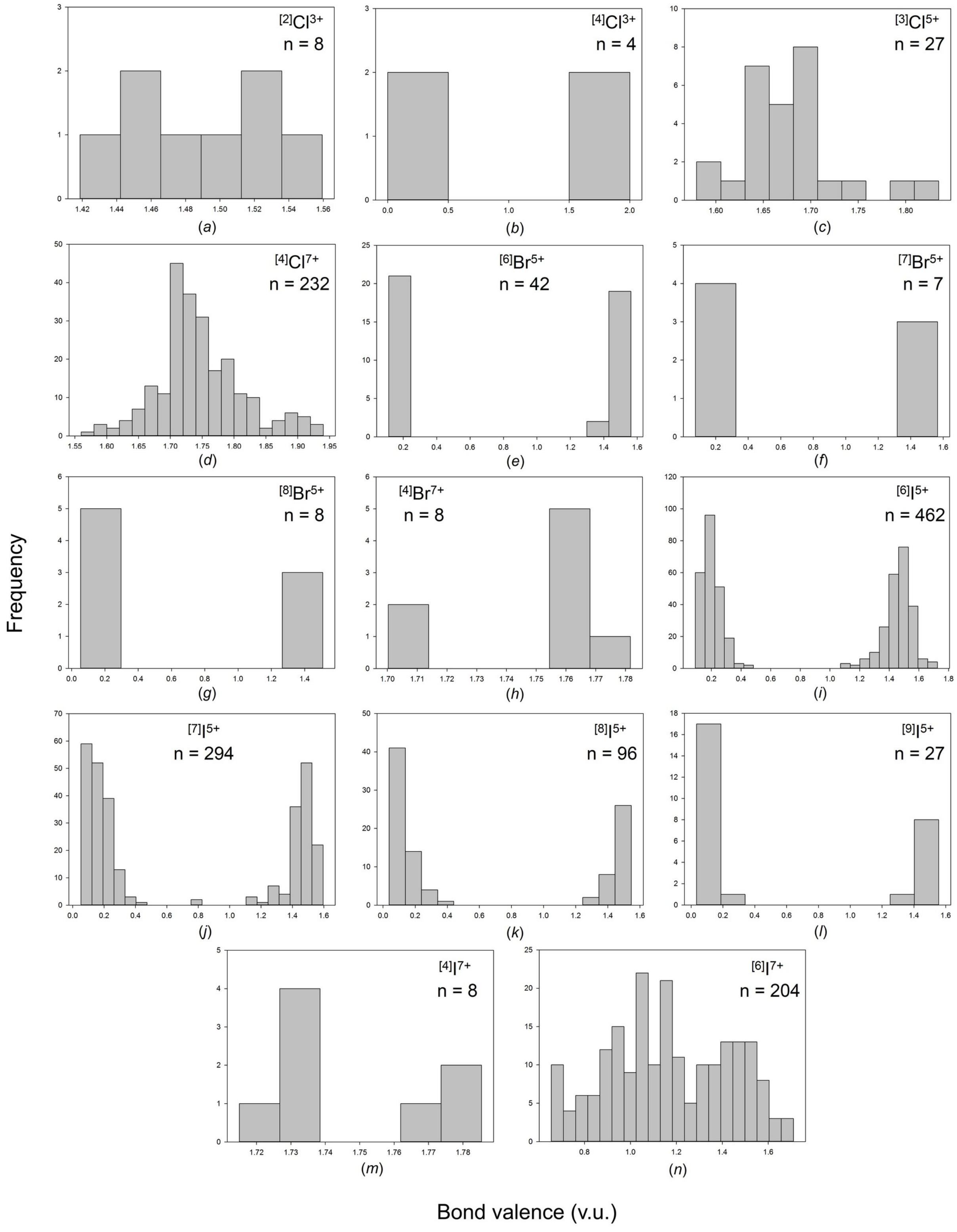
$^{[2]}Cl^{3+}$ n = 8
(a)
$^{[4]}Cl^{3+}$ n = 4
(b)
$^{[3]}Cl^{5+}$ n = 27
(c)
$^{[4]}Cl^{7+}$ n = 232
(d)
$^{[6]}Br^{5+}$ n = 42
(e)
$^{[7]}Br^{5+}$ n = 7
(f)
$^{[8]}Br^{5+}$ n = 8
(g)
$^{[4]}Br^{7+}$ n = 8
(h)
$^{[6]}I^{5+}$ n = 462
(i)
$^{[7]}I^{5+}$ n = 294
(j)
$^{[8]}I^{5+}$ n = 96
(k)
$^{[9]}I^{5+}$ n = 27
(l)
$^{[4]}I^{7+}$ n = 8
(m)
$^{[6]}I^{7+}$ n = 204
(n)
Frequency
Bond valence (v.u.)

Figure S7 Mean-bond-length distributions for all configurations of the hydrogen ion bonded to O^{2-}: (*a*) $^{[2]}H^{+}$, (*b*) $^{[3]}H^{+}$, (*c*) $^{[4]}H^{+}$.

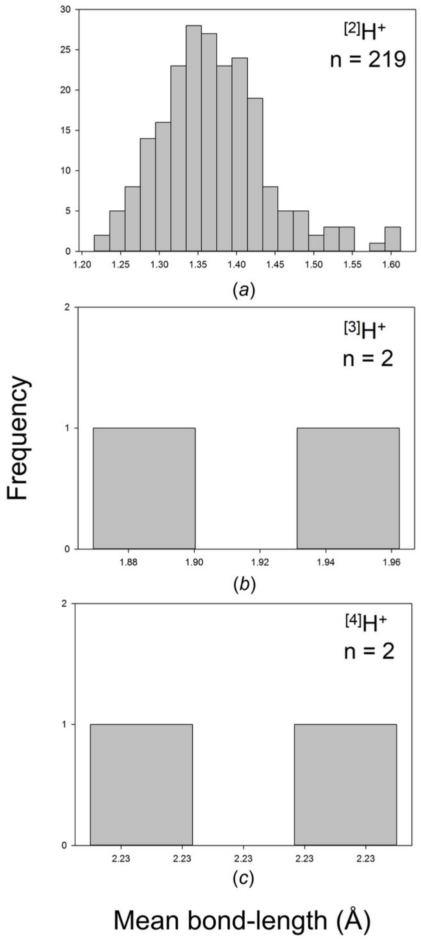
[2]H+
n = 219
[3]H+
n = 2
[4]H+
n = 2
(a)
(b)
(c)
Frequency
Mean bond-length (Å)

Figure S8 Mean-bond-length distributions for all configurations of the group 14-16 non-metal ions bonded to O^{2-}: (*a*) $^{[3]}C^{4+}$, (*b*) $^{[3]}N^{5+}$, (*c*) $^{[4]}N^{5+}$, (*d*) $^{[3]}P^{3+}$, (*e*) $^{[4]}P^{5+}$, (*f*) $^{[3]}S^{4+}$, (*g*) $^{[4]}S^{6+}$, (*h*) $^{[3]}Se^{4+}$, (*i*) $^{[4]}Se^{4+}$, (*j*) $^{[5]}Se^{4+}$, (*k*) $^{[6]}Se^{4+}$, (*l*) $^{[7]}Se^{4+}$, (*m*) $^{[8]}Se^{4+}$, (*n*) $^{[9]}Se^{4+}$, (*o*) $^{[10]}Se^{4+}$, (*p*) $^{[4]}Se^{6+}$.

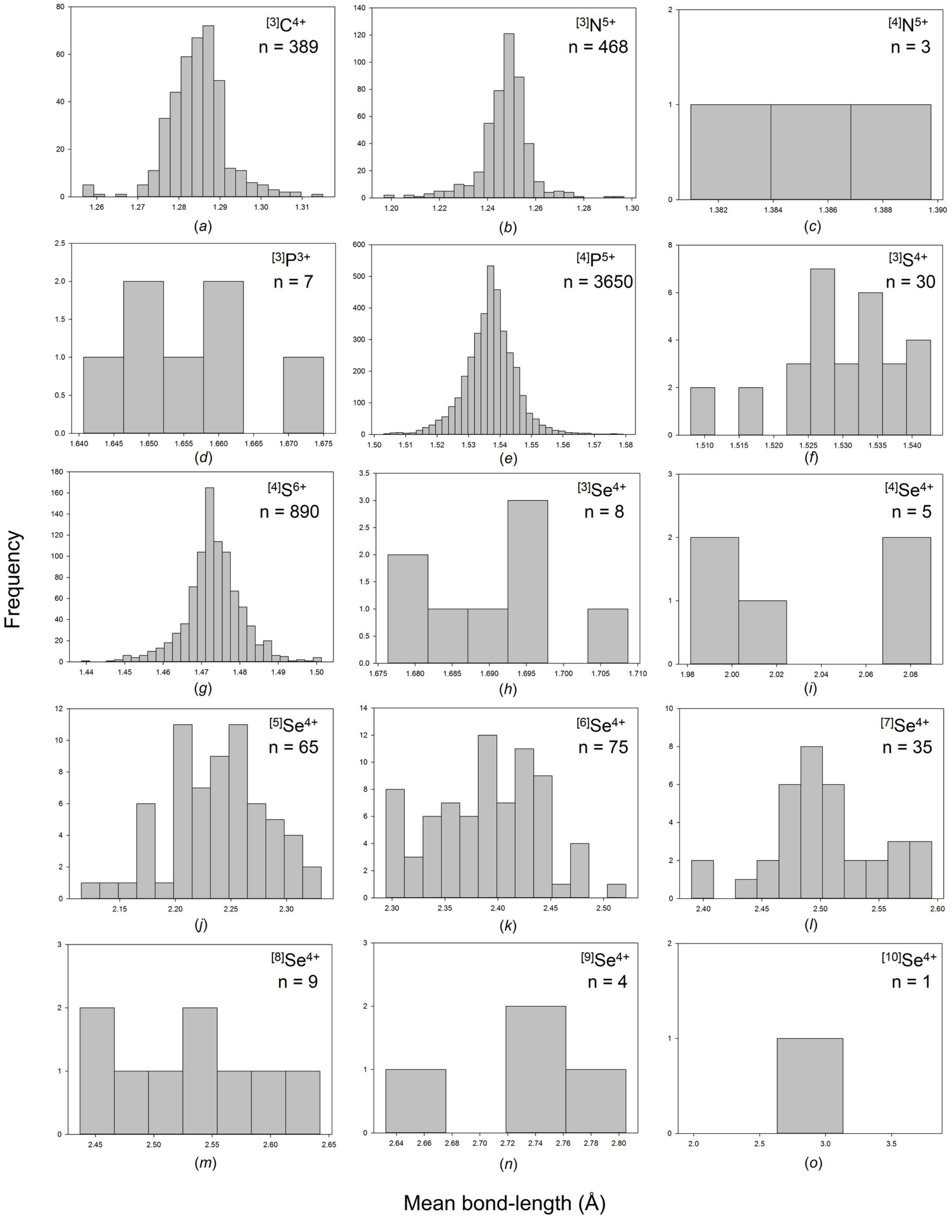
[3]C4+
n = 389
(a)
[3]N5+
n = 468
(b)
[4]N5+
n = 3
(c)
[3]P3+
n = 7
(d)
[4]P5+
n = 3650
(e)
[3]S4+
n = 30
(f)
[4]S6+
n = 890
(g)
[3]Se4+
n = 8
(h)
[4]Se4+
n = 5
(i)
[5]Se4+
n = 65
(j)
[6]Se4+
n = 75
(k)
[7]Se4+
n = 35
(l)
[8]Se4+
n = 9
(m)
[9]Se4+
n = 4
(n)
[10]Se4+
n = 1
(o)
Frequency
Mean bond-length (Å)

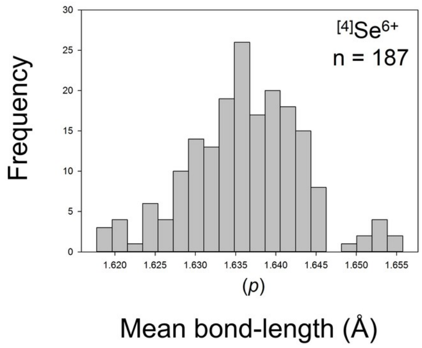

[4]Se6+
n = 187
Frequency
0
5
10
15
20
25
30
1.620
1.625
1.630
1.635
1.640
1.645
1.650
1.655
(p)
Mean bond-length (Å)

Figure S9 Mean-bond-length distributions for all configurations of the group 17 non-metal ions bonded to O^{2-}: (*a*) $^{[2]}Cl^{3+}$, (*b*) $^{[4]}Cl^{3+}$, (*c*) $^{[3]}Cl^{5+}$, (*d*) $^{[4]}Cl^{7+}$, (*e*) $^{[6]}Br^{5+}$, (*f*) $^{[7]}Br^{5+}$, (*g*) $^{[8]}Br^{5+}$, (*h*) $^{[4]}Br^{7+}$, (*i*) $^{[6]}I^{5+}$, (*j*) $^{[7]}I^{5+}$, (*k*) $^{[8]}I^{5+}$, (*l*) $^{[9]}I^{5+}$, (*m*) $^{[4]}I^{7+}$, (*n*) $^{[6]}I^{7+}$.

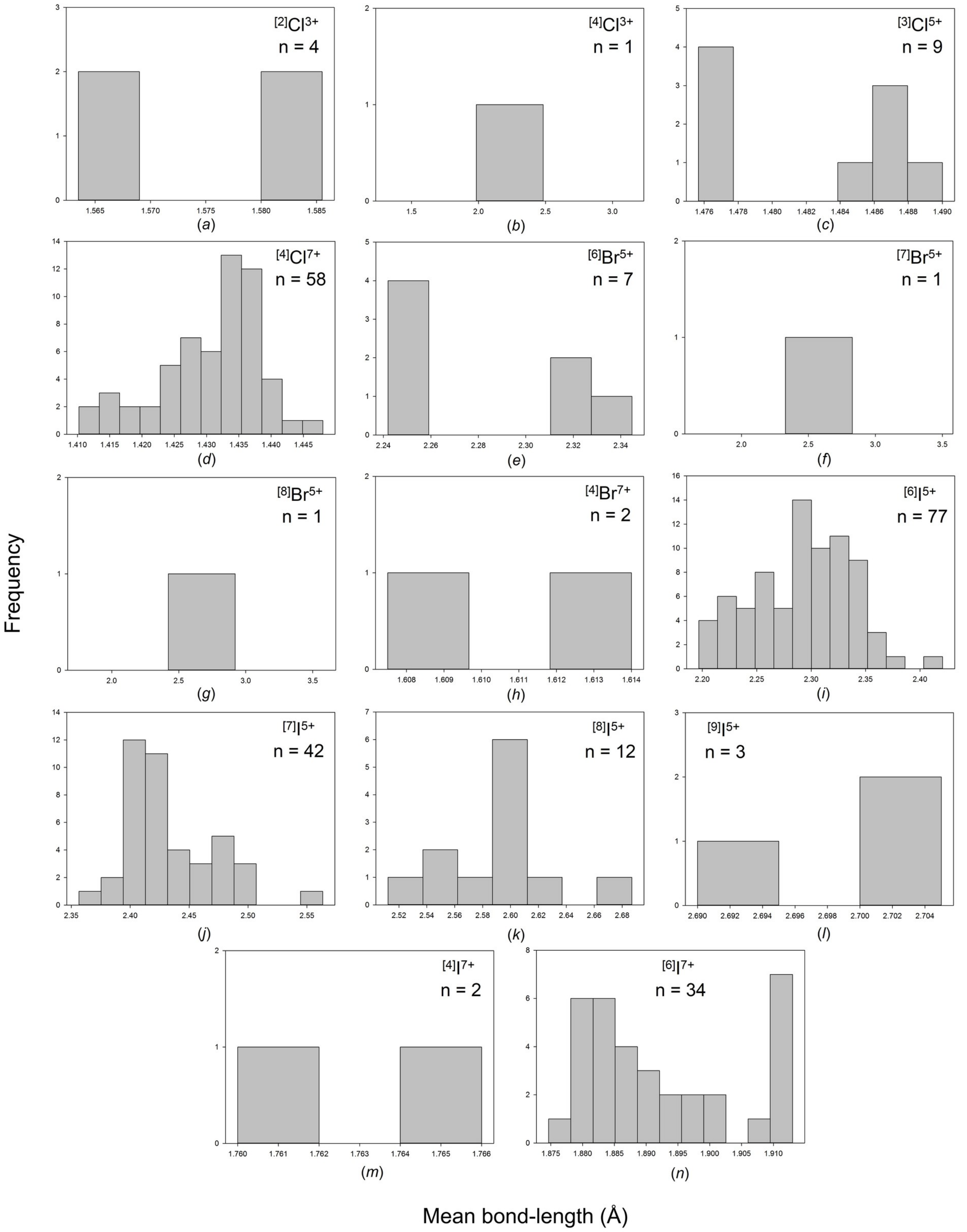
[2]Cl3+
n = 4
(a)
[4]Cl3+
n = 1
(b)
[3]Cl5+
n = 9
(c)
[4]Cl7+
n = 58
(d)
[6]Br5+
n = 7
(e)
[7]Br5+
n = 1
(f)
[8]Br5+
n = 1
(g)
[4]Br7+
n = 2
(h)
[6]I5+
n = 77
(i)
[7]I5+
n = 42
(j)
[8]I5+
n = 12
(k)
[9]I5+
n = 3
(l)
[4]I7+
n = 2
(m)
[6]I7+
n = 34
(n)
Frequency
Mean bond-length (Å)

Figure S10 The effect of bond-length distortion on mean bond-length for all configurations of the hydrogen ion bonded to O^{2-}: (*a*) $^{[2]}H^+$, (*b*) $^{[3]}H^+$, (*c*) $^{[4]}H^+$.

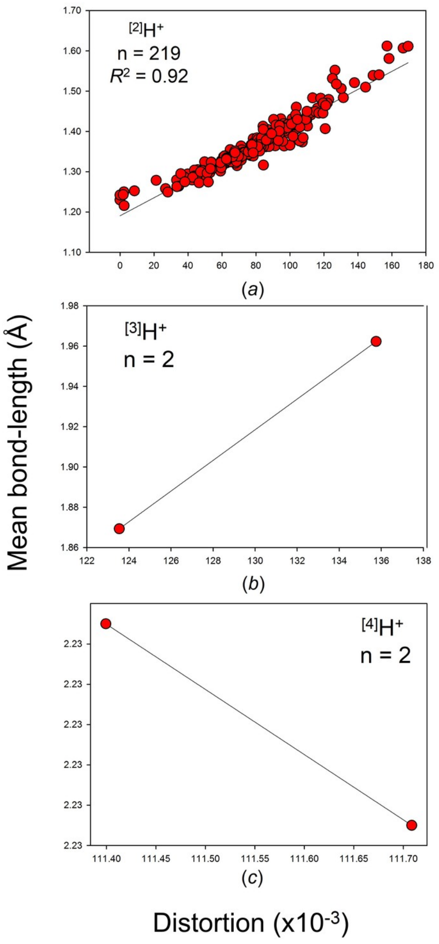
[2]H+
n = 219
R2 = 0.92
(a)
[3]H+
n = 2
(b)
[4]H+
n = 2
(c)
Mean bond-length (Å)
Distortion (x10-3)

Figure S11 The effect of bond-length distortion on mean bond-length for all configurations of the group 14-16 non-metal ions bonded to O^{2-}: (*a*) $^{[3]}C^{4+}$, (*b*) $^{[3]}N^{5+}$, (*c*) $^{[4]}N^{5+}$, (*d*) $^{[3]}P^{3+}$, (*e*) $^{[4]}P^{5+}$, (*f*) $^{[3]}S^{4+}$, (*g*) $^{[4]}S^{6+}$, (*h*) $^{[3]}Se^{4+}$, (*i*) $^{[4]}Se^{4+}$, (*j*) $^{[5]}Se^{4+}$, (*k*) $^{[6]}Se^{4+}$, (*l*) $^{[7]}Se^{4+}$, (*m*) $^{[8]}Se^{4+}$, (*n*) $^{[9]}Se^{4+}$, (*o*) $^{[10]}Se^{4+}$, (*p*) $^{[4]}Se^{6+}$.

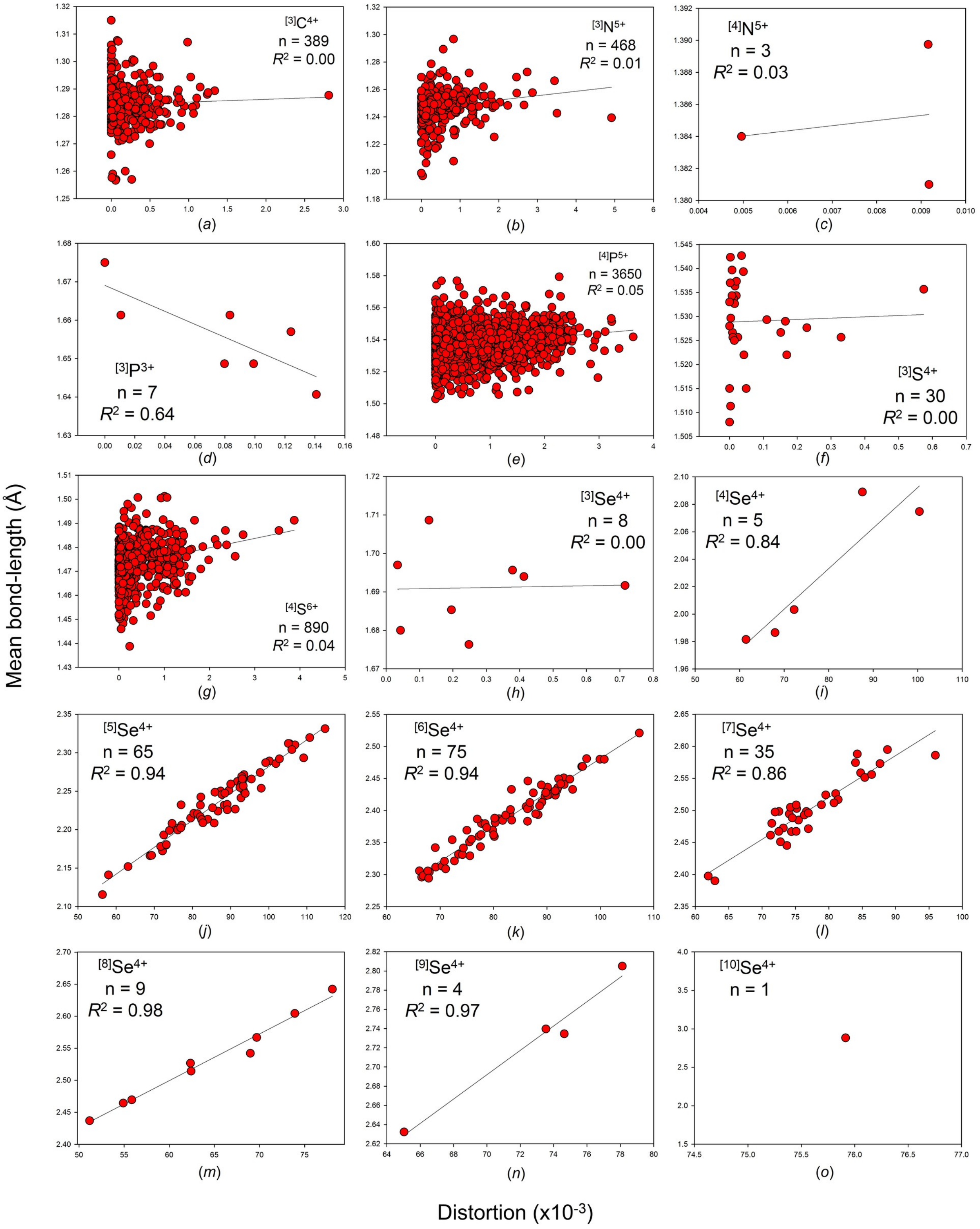
[3]C4+
n = 389
R2 = 0.00
(a)
[3]N5+
n = 468
R2 = 0.01
(b)
[4]N5+
n = 3
R2 = 0.03
(c)
[3]P3+
n = 7
R2 = 0.64
(d)
[4]P5+
n = 3650
R2 = 0.05
(e)
[3]S4+
n = 30
R2 = 0.00
(f)
[4]S6+
n = 890
R2 = 0.04
(g)
[3]Se4+
n = 8
R2 = 0.00
(h)
[4]Se4+
n = 5
R2 = 0.84
(i)
[5]Se4+
n = 65
R2 = 0.94
(j)
[6]Se4+
n = 75
R2 = 0.94
(k)
[7]Se4+
n = 35
R2 = 0.86
(l)
[8]Se4+
n = 9
R2 = 0.98
(m)
[9]Se4+
n = 4
R2 = 0.97
(n)
[10]Se4+
n = 1
(o)
Mean bond-length (Å)
Distortion (x10-3)

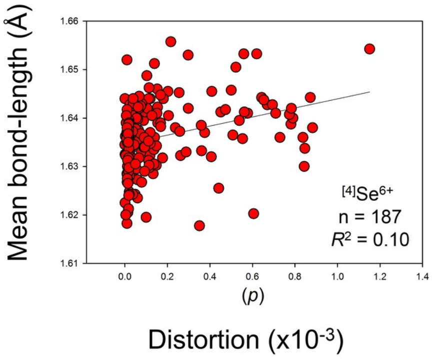
Mean bond-length (Å)
1.66
1.65
1.64
1.63
1.62
1.61
0.0
0.2
0.4
0.6
0.8
1.0
1.2
1.4
[4]Se6+
n = 187
R2 = 0.10
(p)
Distortion (x10-3)

Figure S12 The effect of bond-length distortion on mean bond-length for all configurations of the group 17 non-metal ions bonded to O^{2-}: (*a*) $^{[2]}Cl^{3+}$, (*b*) $^{[4]}Cl^{3+}$, (*c*) $^{[3]}Cl^{5+}$, (*d*) $^{[4]}Cl^{7+}$, (*e*) $^{[6]}Br^{5+}$, (*f*) $^{[7]}Br^{5+}$, (*g*) $^{[8]}Br^{5+}$, (*h*) $^{[4]}Br^{7+}$, (*i*) $^{[6]}I^{5+}$, (*j*) $^{[7]}I^{5+}$, (*k*) $^{[8]}I^{5+}$, (*l*) $^{[9]}I^{5+}$, (*m*) $^{[4]}I^{7+}$, (*n*) $^{[6]}I^{7+}$.

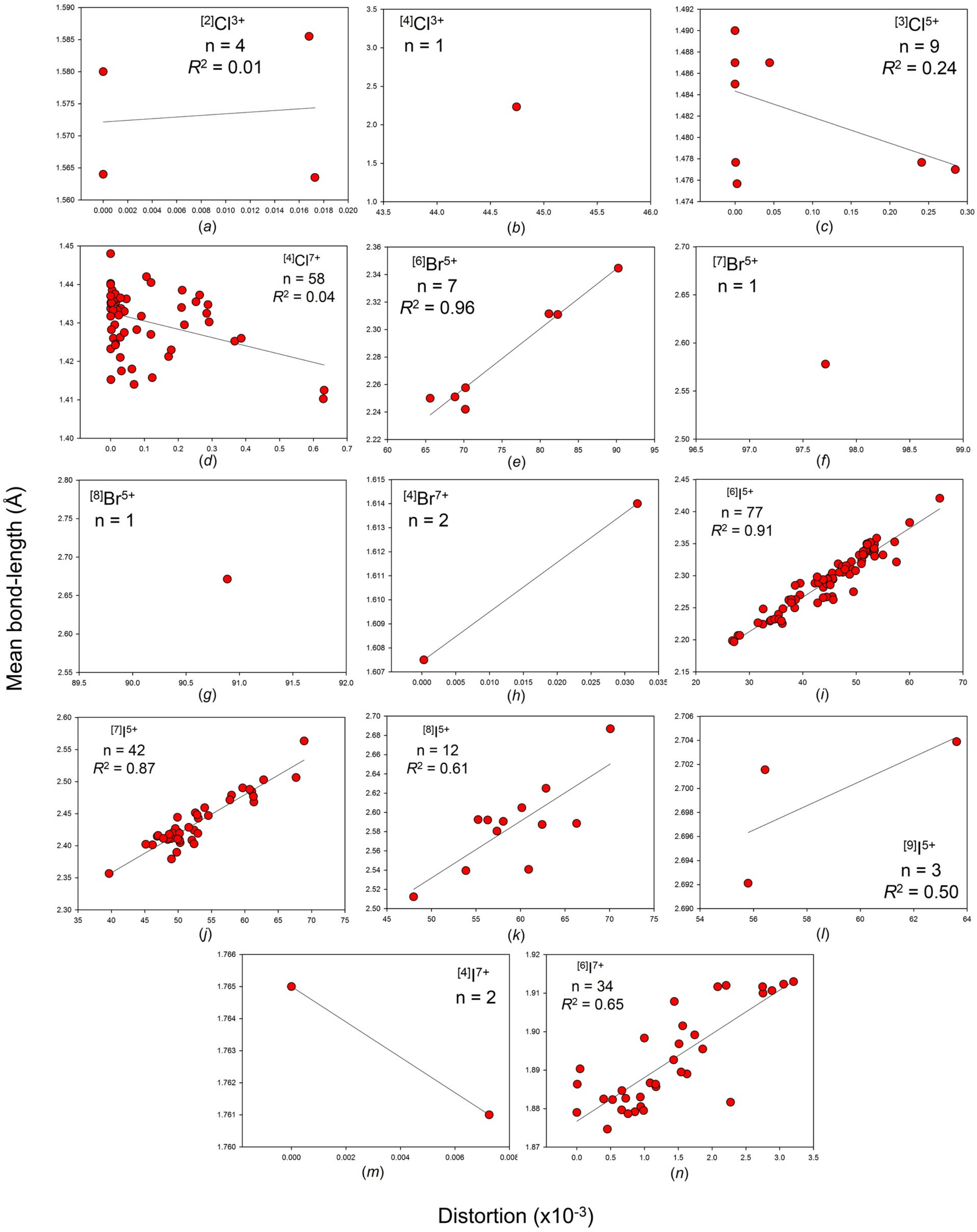

[2]Cl3+
n = 4
R2 = 0.01
(a)
[4]Cl3+
n = 1
(b)
[3]Cl5+
n = 9
R2 = 0.24
(c)
[4]Cl7+
n = 58
R2 = 0.04
(d)
[6]Br5+
n = 7
R2 = 0.96
(e)
[7]Br5+
n = 1
(f)
[8]Br5+
n = 1
(g)
[4]Br7+
n = 2
(h)
[6]I5+
n = 77
R2 = 0.91
(i)
[7]I5+
n = 42
R2 = 0.87
(j)
[8]I5+
n = 12
R2 = 0.61
(k)
[9]I5+
n = 3
R2 = 0.50
(l)
[4]I7+
n = 2
(m)
[6]I7+
n = 34
R2 = 0.65
(n)
Mean bond-length (Å)
Distortion (x10-3)
